# Microstate permutation complexity of EEG signals distinguishes minimally conscious state plus from minimally conscious state minus

**DOI:** 10.1186/s12984-026-01993-w

**Published:** 2026-04-30

**Authors:** Zhibin Zhao, Zhenhu Liang, Yong Wang, Xiaoli Li, He Chen

**Affiliations:** 1 Department of Electrical Engineering, Yanshan University, Qinhuangdao, China; 2 Key Laboratory of Intelligent Rehabilitation and Neuromodulation of Hebei Province, Qinhuangdao, China; 3 Department of Rehabilitation Medicine, Zhujiang Hospital, Southern Medical University, Guangzhou, China; 4 School of Automation Science and Engineering, South China University of Technology, Guangzhou, China; 5 PAZHOU Laboratory (Guangzhou), Guangzhou, China

**Keywords:** Electroencephalography, Disorders of consciousness, Microstate complexity, Lempel-Ziv complexity, Machine learning, Support vector machine, Resting-state EEG

## Abstract

**Background:**

Accurately distinguishing minimally conscious state plus (MCS+) from minimally conscious state minus (MCS–) is critical for prognosis and treatment planning. Microstate analysis decomposes multichannel electroencephalography (EEG) into a sequence of brief, relatively stable scalp electric-field topographies, offering a unique spatiotemporal perspective on brain activity. Yet applications of microstate methods to the assessment of disorders of consciousness remain scarce. Moreover, most state-of-the-art studies focus on characterizing the complexity of microstate sequences, while conventional complexity measures overlook transitions between microstates. To address this gap, we propose Microstate Permutation Lempel–Ziv Complexity (MS-PLZC), an extension of Lempel–Ziv complexity that explicitly encodes ordinal permutation information to more sensitively capture the temporal organization of microstate sequences.

**Methods:**

Resting-state EEG was recorded from 45 individuals with disorders of consciousness (15 unresponsive wakefulness syndrome, 15 MCS–, 15 MCS+) and 15 neurologically healthy controls. MS-PLZC, conventional microstate LZC, spectral power, sample entropy, and classical LZC were calculated and statistically compared. These features were assessed using a nested leave-one-out cross-validated (LOOCV) SVM with exhaustive hyper-parameter search.

**Results:**

Both MS-LZC and MS-PLZC showed statistically significant group differences (Kruskal-Wallis test: MS-LZC: H = 26.92, *p* < 0.0000, η²=0.2099; MS-PLZC: H = 35.11, *p* < 0.0000, η²=0.2816), with MS-PLZC exhibiting greater statistical power. Notably, MS-PLZC successfully distinguished between MCS- and MCS+ patients (p _adj < 0.05) with a large effect size (Cliff’s Delta = -0.6178), whereas MS-LZC demonstrated only a medium effect size (Cliff’s Delta = -0.3067). In the machine-learning analysis MS-PLZC achieved the highest leave-one-out accuracy (0.733) and ROC-AUC (0.733).

**Conclusions:**

These results indicate that MS-PLZC sensitively captures subtle shifts in microstate dynamics and offers a reliable single-feature discriminator of MCS+ versus MCS–, with translational potential for detecting key recovery windows during routine assessment of consciousness.

**Supplementary Information:**

The online version contains supplementary material available at 10.1186/s12984-026-01993-w.

## Background

 Disorders of consciousness (DOC) generally stem from structural or functional lesions within the neural circuits that sustain wakefulness and conscious awareness [1–3]. After a severe brain insult, affected individuals often lapse into coma—a state marked by closed eyes and a complete absence of purposeful behavior or subjective experience [4]. Upon awakening, some remain unresponsive to external stimuli—diagnosed as vegetative state/unresponsive wakefulness syndrome (VS/UWS) [5]. Others exhibit fluctuating but clear behavioral signs demonstrating awareness of the self or surroundings, thus meeting criteria for minimally conscious state (MCS) [6]. MCS is further subdivided into minimally conscious state plus (MCS+) and minimally conscious state minus (MCS–), with MCS+ denoting higher-level behavioral outputs such as command following or intelligible speech [7, 8]. While some individuals ultimately regain full consciousness and can reintegrate into everyday life, many persist in prolonged impaired states, causing substantial emotional and financial burdens.

Accurate diagnosis is essential for guiding clinical decisions and detecting early recovery indicators. It also informs the selection and timing of rehabilitation and arousal-promoting interventions, the allocation of clinical resources, and goals-of-care discussions with families. For example, candidacy for invasive neuromodulatory approaches (e.g., spinal cord stimulation) is often considered more appropriate when there is evidence of minimal consciousness, whereas VS/UWS is approached more cautiously given uncertain benefit and high long-term care burden. The JFK Coma Recovery Scale-Revised (CRS-R) is presently viewed as the benchmark tool for distinguishing VS/UWS from MCS [9]. Nonetheless, its performance may be hampered by inter-patient variability and subjective judgment, resulting in misdiagnosis rates of up to 40% [10, 11]. To address these issues, neuroimaging and neurophysiological methods—especially quantitative electroencephalography (qEEG)—have gained prominence [12–16]. Compared with traditional visual assessment, qEEG offers more objective numerical analysis.

Beyond bedside behavioral examination, multiple brain-based approaches have been explored to improve differentiation of VS/UWS and MCS. Metabolic and network neuroimaging (e.g., FDG-PET and resting-state fMRI) has been reported to provide strong complementary evidence for identifying MCS, particularly when behavioral assessment is challenging (e.g., high sensitivity of FDG-PET for MCS in clinical validation studies) [17]. Perturbation–response paradigms combining TMS with EEG, such as the Perturbational Complexity Index (PCI), quantify the brain’s integrated and complex response to external perturbation and have been widely discussed as sensitive markers of residual consciousness [18]. In parallel, bedside EEG remains highly attractive for translation due to its low cost and portability; recent cross-site machine-learning frameworks (e.g., DOC-Forest) suggest that multivariate EEG markers can generalize across centers and protocols with AUC values around 0.75, with alpha/theta dynamics and complexity contributing substantially to discrimination [19]. Moreover, multimodal strategies (e.g., FDG-PET combined with EEG-based classification) have been shown to improve diagnostic performance compared with either modality alone, supporting the value of complementary evidence integration in DoC evaluation [20].

Microstate analysis, which is a new method of qEEG [21], offers a unique spatiotemporal perspective on brain activity by viewing multi-channel EEG as rapidly shifting periods of stable electric field topographies [22, 23]. Each microstate persists for tens to hundreds of milliseconds, after which it shifts abruptly into a different configuration [24, 25]. Such microstate features can reveal dynamic patterns of brain activity crucial for understanding consciousness and its disorders. Moreover, Lempel–Ziv Complexity (LZC) offers a way to evaluate how often novel patterns arise and how unpredictable they are within a time series, thereby serving as a valuable tool for assessing the complexity of microstate sequences [26]. This serves as a metric for assessing the spatiotemporal complexity of EEG. Recent work indicates that spatiotemporal complexity may better capture consciousness levels than average temporal complexity alone [27], and LZC analyses of EEG microstates further highlight changes in different states of consciousness [28].

However, traditional LZC analyses overlook important permutation patterns in microstate sequences [28–33]. To address this gap, we propose an innovative complexity measure—microstate permutation Lempel–Ziv Complexity (MS-PLZC)—that evaluates both the occurrence and ordering of microstate sequences. In this study, we analyzed 45 DOC patients (15 VS/UWS, 15 MCS-, and 15 MCS+) and compared them with 15 healthy controls (CTRL). We assessed frequency power and complexity metrics (e.g., ApEn, SampEn, and LZC), and compared the conventional microstate LZC with our proposed PLZC approach to validate its effectiveness and potential advantages in distinguishing between MCS + and MCS-.

## Materials and methods

### Participants

We included 45 patients with DOC, comprising 15 with UWS, 15 with MCS–, and 15 with MCS+, alongside 15 CTRL. The protocol complied with the Declaration of Helsinki and received approval from the Ethics Committee of the Seventh Medical Center, Chinese PLA General Hospital (approval 2016-63; 31 Oct 2016). Written informed consent was secured from each participant or, when required, from a legal guardian. Healthy controls (7 males, 8 females) were aged 37–57, with no history of epilepsy or recent sedative use. DOC patients (32 males, 13 females) ranged from 15 to 77 years and were medically stable; data collection was halted if their condition changed (e.g., increased muscle tension or persistent teeth clenching). Detailed demographic and clinical data appear in Table 1. Across the four groups, age and sex distributions were comparable (*P* = 0.309 and *P* = 0.139, respectively). Among the patient groups, time since injury was also comparable (*P* = 0.631), whereas aetiology distributions differed (*P* = 0.010); this was therefore considered when interpreting the results.

**Table 1:** Demographic and clinical characteristics of study participants

Variable	UWS	MCS-	MCS+	CTRL	*P* value
Age, years (mean ± SD)	43.13 ± 15.32	46.00 ± 17.39	40.87 ± 14.88	47.93 ± 5.30	0.309†
Sex (male/female), n	13/2	9/6	10/5	7/8	0.139‡
Aetiology (TBI/stroke/hypoxia), n (%)	1 (6.67) / 8 (53.33) / 6 (40.00)	4 (26.67) / 6 (40.00) / 5 (33.33)	10 (66.67) / 4 (26.67) / 1 (6.67)	–	0.010§
Time since injury, (median [IQR])	2.50 [2.00–4.50]	2.00 [1.50–4.25]	2.00 [1.50–4.75]	–	0.631¶
CRS-R total score (median [IQR])	5.00 [4.50–6.00]	8.00 [8.00–10.00]	11.00 [9.00–12.50]	–	–

*VS/UWS* vegetative state/unresponsive wakefulness syndrome, *MCS* minimally conscious state, *CRS-R* JFK Coma Recovery Scale–Revised, *SD* standard deviation, *IQR* interquartile range, *TBI* traumatic brain injury, *CRS-R* is a classification-related clinical scale; therefore, between-group differences are inherent to group definition and are typically not treated as baseline comparability variables (P values are not reported)

† Welch one-way ANOVA across four groups

‡ Chi-square test across four groups

§ Chi-square test across patient groups only (VS/UWS, MCS-, MCS+); not applicable to healthy controls

¶ Kruskal–Wallis test across patient groups only (VS/UWS, MCS-, MCS+)

### Data collection methods: CRS-R and EEG recording

All DOC participants completed three baseline examinations with the CRS-R, each administered by a board-certified neurosurgeon. The CRS-R contains 23 items grouped into five domains—arousal, auditory, visual, motor, and oromotor/verbal function. Behaviors such as eye tracking, sound localisation, and other stimulus-driven responses are pivotal for diagnosing MCS. Each session lasted roughly one hour; the highest score obtained across the three wakeful assessments was retained as the patient’s final CRS-R value.

Resting EEG was then recorded for 10 min using a 32-channel Nicolet V32 amplifier (Natus Neurology) with Ag/AgCl scalp electrodes positioned according to an extended 10–20 montage (10–10 system subset) (see Fig. Sx for the electrode layout) and sampled at 500 Hz. FCz served as the common reference, AFz as ground, and electrode–skin impedance was kept below 5 kΩ throughout acquisition.

### EEG signal preprocessing

Raw EEG data were pre-processed offline in EEGLAB 2023.1 running on MATLAB R2024b (MathWorks, Natick, MA, USA). The preprocessing pipeline consisted of the following steps: (1) Resting-state EEG was acquired continuously; segments with obvious noise (e.g., large movement-related drifts, sustained EMG bursts, or electrode artifacts) were visually identified and removed, and the remaining clean segments were then concatenated to form a continuous time series for subsequent analyses; bad channels were interpolated when necessary. (2) Mains interference at 50 Hz was removed with EEGLAB’s CleanLine plugin, followed by band-pass filtering the signals from 1 to 45 Hz. (3) The signals were subsequently resampled to 250 Hz. (4) Ocular and muscle artifacts were eliminated via independent component analysis (ICA) using the runica algorithm (extended Infomax); ICLabel (default) was applied to assist component classification, and components were removed after visual inspection of scalp topographies, time courses, and power spectra. (5) Re-referenced using the average reference.

After preprocessing (manual rejection, ICA cleaning, and concatenation), the retained clean EEG duration (mean ± SD, min–max; in seconds) was: CTRL 595.00 ± 14.11 (545.32–600.00), MCS + 595.20 ± 9.78 (569.30–600.00), MCS − 575.91 ± 78.08 (299.41–600.01), and VS/UWS 599.65 ± 0.96 (596.83–600.00); thus, the minimal retained clean duration was 299.41 s (≈ 5.0 min). The amount of manually rejected data (seconds; mean ± SD) was: CTRL 15.22 ± 20.05, MCS + 115.31 ± 119.15, MCS − 123.27 ± 124.19, and VS/UWS 136.92 ± 218.02. The average number of removed ICA components (mean ± SD) was: CTRL 7.40 ± 2.16, MCS + 4.80 ± 2.18, MCS − 5.00 ± 3.34, and VS/UWS 4.87 ± 2.97.

### EEG signal analysis

**Fig. 1:**
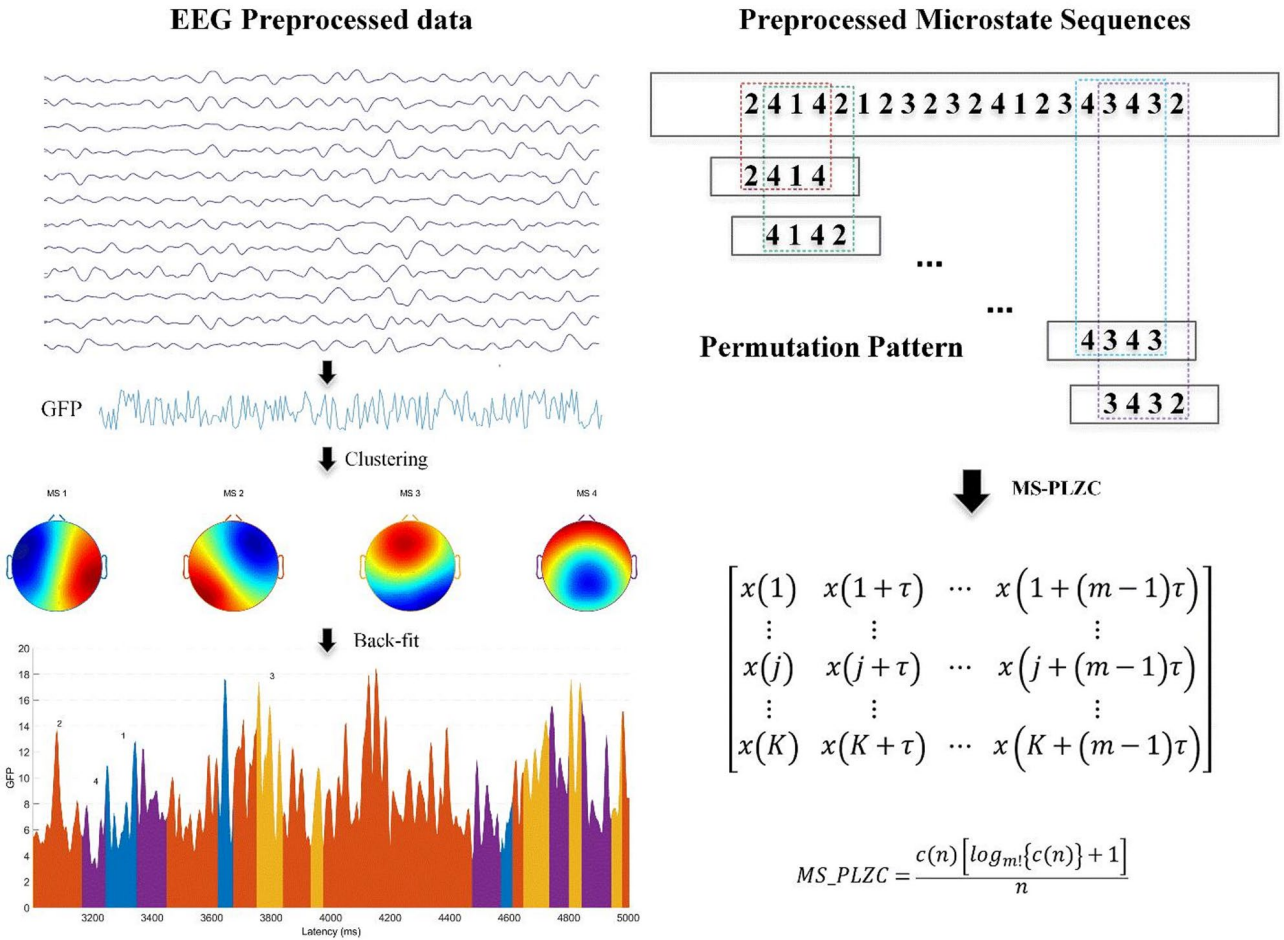
EEG feature analysis framework for MS-PLZC in DOC patients

Figure 1 illustrates the overall workflow of MS-PLZC. First, the EEG signals are preprocessed and segmented into microstates, after which microstate sequences are extracted. Finally, LZC and PLZC are applied to assess the spatiotemporal complexity of these microstate sequences. Since this paper focuses primarily on microstate complexity features, the complete calculation methods for microstate duration, coverage, occurrence, and transition probabilities can be found in the Supplementary Materials. Other EEG-based features (including relative power and temporal complexity), which are mainly used to compare performance with MS-PLZC in distinguishing consciousness states, are also detailed in the Supplementary Materials.

### Extraction of microstates

EEG microstates are transient intervals of about 80–120 ms in which the scalp voltage map remains nearly stable, reflecting the brain’s fast sub-second spatiotemporal dynamics. This illustrates the rapid spatiotemporal unfolding of neural activity on a sub-second timescale. Microstate analysis converts multichannel EEG signals into microstate prototypes. The resulting global field power (GFP) trace illustrates the temporal evolution of overall field strength. Local maxima—moments of maximal scalp-potential amplitude—are identified as points of quasi‐stable topographic configuration. These peak intervals are then extracted and clustered to derive the final set of stable microstate prototypes.

Dominant microstate classes were extracted with a refined K-means clustering approach. Compared with the standard implementation, this variant converges more rapidly and yields higher partition accuracy in the presence of noise, outliers, or clusters with disparate medians. The algorithm initializes by randomly selecting k prototype maps (k = 2 ~ 8 in this study). At each iteration, the Global Explained Variance (GEV) is recomputed until it plateaus, and the resulting prototypes are then adopted as the final microstate templates.

Accurate microstate quantification is critical for downstream analyses. Therefore, we assessed the microstate model with two polarity-invariant indices: GEV and the cross-validation (CV) metric. GEV gauges how closely each EEG topography aligns with its allocated microstate template, with higher values indicating superior clustering quality. It is defined as follows:

1 $$GEV=\frac{{{{\left( {Corr\left( {{x_n},{a_{ln}}} \right) \cdot GF{P_n}} \right)}^2}}}{{\mathop \sum \nolimits_{n}^{N} GFP_{n}^{2}}}$$

where $\:n$ indexes each sample; $\:{GFP}_{n}$ is the Global Field Power at sample $\:n$; xn denotes the EEG data vector at time $\:n$; $\:{a}_{ln}$ is the microstate label assigned to that sample; $\:Corr({x}_{n},{a}_{ln})$ is the Pearson correlation between the data vector and its corresponding template; where $\:N$ denotes the total number of observations.

The CV metric quantifies residual variance and noise in the microstate model. Poorly fitting solutions produce larger residuals, so lower CV scores indicate more reliable clustering. CV is defined as:

2 $$CV={\hat {\sigma }^2} \cdot {\left( {\frac{{C - 1}}{{C - K - 1}}} \right)^2}$$

Here, $\:C$ is the number of EEG channels, $\:K$ is the number of microstate clusters, and $\:{\widehat{\sigma\:}}^{2}$ denotes the estimated variance of the residual noise.

### Microstate complexity analysis

1. Microstate Sequence Preprocessing.

Sequences were segmented into contiguous “chunks,” each comprising consecutive repetitions of a single microstate (A–F)—for example, [AABCCC] yields three chunks (AA, B, CCC).

- Removal of unlabeled samples. Any NaN or otherwise unlabeled samples were discarded (e.g., [AAB NaN CCC] → [AABCCC]).
- Short-chunk relabeling. Chunks of length ≤ 4 samples were replaced by new chunks of identical length carrying the label of the nearest long chunk, with preference given to the preceding chunk (or the following one if no preceding chunk existed).
- Long-chunk stabilization. Chunks of length ≥ 5 samples (e.g., [AAAABCDDDDD]) were truncated to remove unstable portions, retaining only the central stable segments (e.g., [AAAAAAA DDDDD]), thereby excluding microstates whose duration fell below 16ms.
- Symbolic collapse. Each stabilized chunk was then collapsed to a single symbol, yielding a “non-durable” sequence that omits consecutive repeats (e.g., [AAAAAACCCCCBBBBB] → [ACB]).

(2) Microstate Lempel-Ziv Complexity (MS-LZC).

LZC is widely employed to capture the spatiotemporal intricacy of EEG activity. Classical LZC computation first binarises and detrends the data. In this study, MS-LZC is computed as the byte size of the compressed microstate symbol sequence. For every participant and experimental condition, MS-LZC was evaluated on successive 5-s windows (≈ 150 microstates) extracted from the full non-durable sequence with 90% overlap, yielding a smooth and representative complexity profile.

LZC increases with signal randomness. We calculated LZC for each microstate sequence and label this metric as MS-LZC.

(3) Microstate Permutation-Lempel-Ziv Complexity.

For the microstate series $\:{\left\{X\left(i\right)\right\}}_{i=1}^{n}$, we embed it in phase space to construct the following matrix:

$$\left[ {\begin{array}{*{20}{c}} {x\left( 1 \right)}&{x\left( {1+\tau } \right)}& \cdots &{x\left( {1+\left( {m - 1} \right)\tau } \right)} \\ \vdots & \vdots &~& \vdots \\ {x\left( j \right)}&{x\left( {j+\tau } \right)}& \cdots &{x\left( {j+\left( {m - 1} \right)\tau } \right)} \\ \vdots & \vdots &~& \vdots \\ {x\left( K \right)}&{x\left( {K+\tau } \right)}& \cdots &{x\left( {K+\left( {m - 1} \right)\tau } \right)} \end{array}} \right]$$

  

$$j=1,2, \cdots ,K$$

where: $\:m$ specifies the embedding dimension and $\:\tau\:$ the time delay; $\:K=n-\left(m-1\right)\tau\:$. Each row in the matrix can be considered as a reconstructed component, with $\:K$ reconstructed components in total.

In the present work, we first encoded the sequence patterns into a symbolic string and then performed LZC computation on this representation. These patterns are also known as motifs [34]. Since, in the preprocessed microstate sequence, two adjacent microstates are not the same, the total count of admissible motifs can be written as $\:m\times\:\:{(m-1)}^{3}$, where m simultaneously denotes the motif length and the number of microstate categories. When $\:m=4$, each motif therefore contains four microstates, yielding 108 distinct motif combinations.

The lag parameter $\:\tau\:$ specifies the interval—measured in sample points—between successive entries within a motif. With this definition, the microstate series is re-coded as a symbolic string whose alphabet consists of the integers 1–108, each number uniquely identifying a motif. Two parameters, $\:m$ and $\:\tau\:$, dominate this permutation framework; varying either one produces a different symbolic sequence. Since the number of our microstates is 4, $\:m=4$. Because we need to measure all permutation patterns of microstates, we choose $\:\tau\:=1$ to cover all microstate sequences.

For example, starting with the first state as ms1, the possible permutation patterns are $\:3\times\:3\times\:3=27$ kinds:

$$ \left[ {\begin{array}{*{20}c} ~ & ~ & {ms1} & {ms2/ms3/ms4} \\ ~ & {ms2} & {ms3} & {ms1/ms2/ms4} \\ ~ & ~ & {ms4} & {ms1/ms2/ms3} \\ ~ & ~ & ~ & ~ \\ ~ & ~ & {ms1} & {ms2/ms3/ms4} \\ {ms1} & {ms3} & {ms2} & {ms1/ms3/ms4} \\ ~ & ~ & {ms4} & {ms1/ms2/ms3} \\ ~ & ~ & ~ & ~ \\ ~ & ~ & {ms1} & {ms2/ms3/ms4} \\ ~ & {ms4} & {ms2} & {ms1/ms3/ms4} \\ ~ & ~ & {ms3} & {ms1/ms2/ms4} \\ \end{array} } \right] $$

  

Each row in the reconstructed matrix $\:X\left(i\right)$ is considered as one of the arrangement patterns of the microstate sequence. The computation proceeds through the following steps:

- Step 1 | Symbolic encoding: Transform the microstate series into a finite symbolic sequence $\:\left\{x\right(n\left)\right\}$. Under this encoding the total number of permissible permutation patterns does not exceed $\:m\times\:\:{(m-1)}^{3}$.
- Step 2 | Initialization: Let $\:S$ be the first symbol of $\:\left\{x\right(n\left)\right\}$, $\:Q$ the second, and set the complexity counter $\:\mathrm{c}\left(\mathrm{n}\right)=1$.
- Step 3 | Concatenation and truncation: Merge $\:S$ and $\:Q$ to obtain $\:SQ$. Define $\:SQv$ as $\:SQ$ with its last symbol removed. For example, if:

$$\:S={x}_{1},\dots\:,{x}_{i},$$

$$\:Q={x}_{i+1},\dots\:,{x}_{i+j-1},$$

- then:

$$\:SQ={x}_{1},\dots\:,{x}_{i+j},$$

$$\:SQv={x}_{1},\dots\:,{x}_{i+j-1}.$$

- Step 4 | Termination check: If $\:Q$ already includes the final symbol of $\:\left\{x\right(n\left)\right\}$, skip to Normalization; otherwise continue.
- Step 5 | Substring match: Enumerate every substring of $\:SQv$ to form a vocabulary $\:V$.

- If $\:Q\in\:V$, proceed to Step 6.
- Otherwise $\:Q$ represents a new pattern; go to Step 7.

- Step 6 | Extend $\:Q$: Append the next symbol in $\:\left\{x\right(n\left)\right\}$ to $\:Q$ and return to Step 3.
- Step 7 | Update and count: Update $\:S$ to $\:SQ$, let $\:Q$ equal the next symbol of $\:\left\{x\right(n\left)\right\}$, and increment $\:c\left(n\right)$ by 1. Return to Step 3.
- Step 8 | Complexity and normalization: After the entire sequence has been scanned, $\:c\left(n\right)$ is the empirical complexity of $\:\left\{x\right(n\left)\right\}$. The maximal number of distinct substrings is bounded by [35]:

3 $$L\left( n \right)=c\left( n \right)\left[ {lo{g_{m!}}\left\{ {c\left( n \right)} \right\}+1} \right]$$

The MS-PLZC is then normalized as:

4 $$MS\_PLZC=\frac{{c\left( n \right)\left[ {lo{g_{m!}}\left\{ {c\left( n \right)} \right\}+1} \right]}}{n}$$

where $\:n$ is the length of the symbolic sequence.

For sufficiently large $\:n$, Eq. (4) simplifies to:

5 $$MS\_PLZC=\frac{{c\left( n \right)\left\{ {lo{g_{m!}}n} \right\}}}{n}$$

### Statistical analysis

All statistical procedures were executed via custom R scripts in RStudio 2023.03.1. When comparing more than two groups, we applied the Kruskal–Wallis test whenever either the normality assumption or equality of variances was violated; if these assumptions were satisfied, a one-way ANOVA was instead used. Pairwise follow-up comparisons employed the Mann–Whitney U test for non-parametric data and the independent-samples t test when parametric assumptions were satisfied. Where relevant, effect sizes were provided—Eta-squared ($$ \:\eta ^{2} $$) for Kruskal–Wallis results and Cliff’s Delta for pairwise tests. Family-wise error was controlled via a Bonferroni correction, maintaining the significance threshold at 0.05. Statistical significance is denoted as follows: $$\:*\:p\:\_adj\:<\:0.05,\:**\:p\:\_adj\:<\:0.01,\:and\:1$$$$***\:p\:\_adj\:<\:0.00$$

### Machine learning

In this study, 13 candidate feature sets were extracted from resting-state EEG as inputs to the machine-learning model: (i) seven relative power ratios—$\:\delta\:$, $\:\theta\:$, $\:\alpha\:$, $\:\beta\:$, $\:\gamma\:$, $\:(\alpha\:+\beta\:)/(\delta\:+\theta\:)$, and $\:\alpha\:/\beta\:$; (ii) two classical nonlinear metrics (Lempel–Ziv complexity and SampEn); (iii) three microstate-based complexity measures, including the MS-PLZC proposed herein; and (iv) two compound sets, Power-7 and NL-2. These yielded a 30 × 11 feature matrix (15 MCS- and 15 MCS+) that was z-scored column-wise (mean = 0, SD = 1).

Classification was carried out with the C-support vector classification (C-SVC) implementation of libsvm-3.25. Four kernel families were systematically explored—linear, second- and third-order polynomial, radial basis function (RBF), and sigmoid. A coarse grid was first defined in log₂ space ($C \in \hat{2} - ^{{7:3:17}}$ and γ ∈ $\gamma \in \hat{2} - ^{{17:3:5}}$; polynomial kernels additionally searched d = 2 or 3). Around the best coarse point, a finer grid with a 0.5 log₂ step was deployed, resulting in 441–882 parameter combinations per feature set. To prevent information leakage and obtain an almost unbiased estimate of generalization performance, we adopted a strictly nested leave-one-out cross-validation (LOOCV) scheme: in the outer loop each subject was left out as the test case, while the remaining 29 formed the training set; within this training subset a five-fold inner CV selected the optimal (kernel, C, γ, d), after which the model was retrained on all 29 samples and applied to the held-out subject.

From the 30 outer predictions we computed accuracy, sensitivity, and specificity. Decision values were direction-corrected, and the area under the receiver operating characteristic curve (ROC-AUC) was obtained via trapezoidal integration. The kernel and hyper-parameters most frequently selected across the 30 outer iterations were reported as the representative model for each feature set, and all fold-specific optimal parameters were retained in full. All analyses were implemented in MATLAB R2024b.

## Results

### Microstate topology

When analyzing the GFP time-series, we employed a modified K-means procedure that disregards the polarity of EEG topographic maps. The number of microstate prototypes was restricted to the range 2–8 for the initial clustering. Resulting prototypes were subsequently ranked by their global explained variance. After partitioning the GFP sequence into candidate microstates, we identified the optimal cluster count for subsequent analyses—a prerequisite for meaningful, in-depth interpretation.

**Fig. 2:**
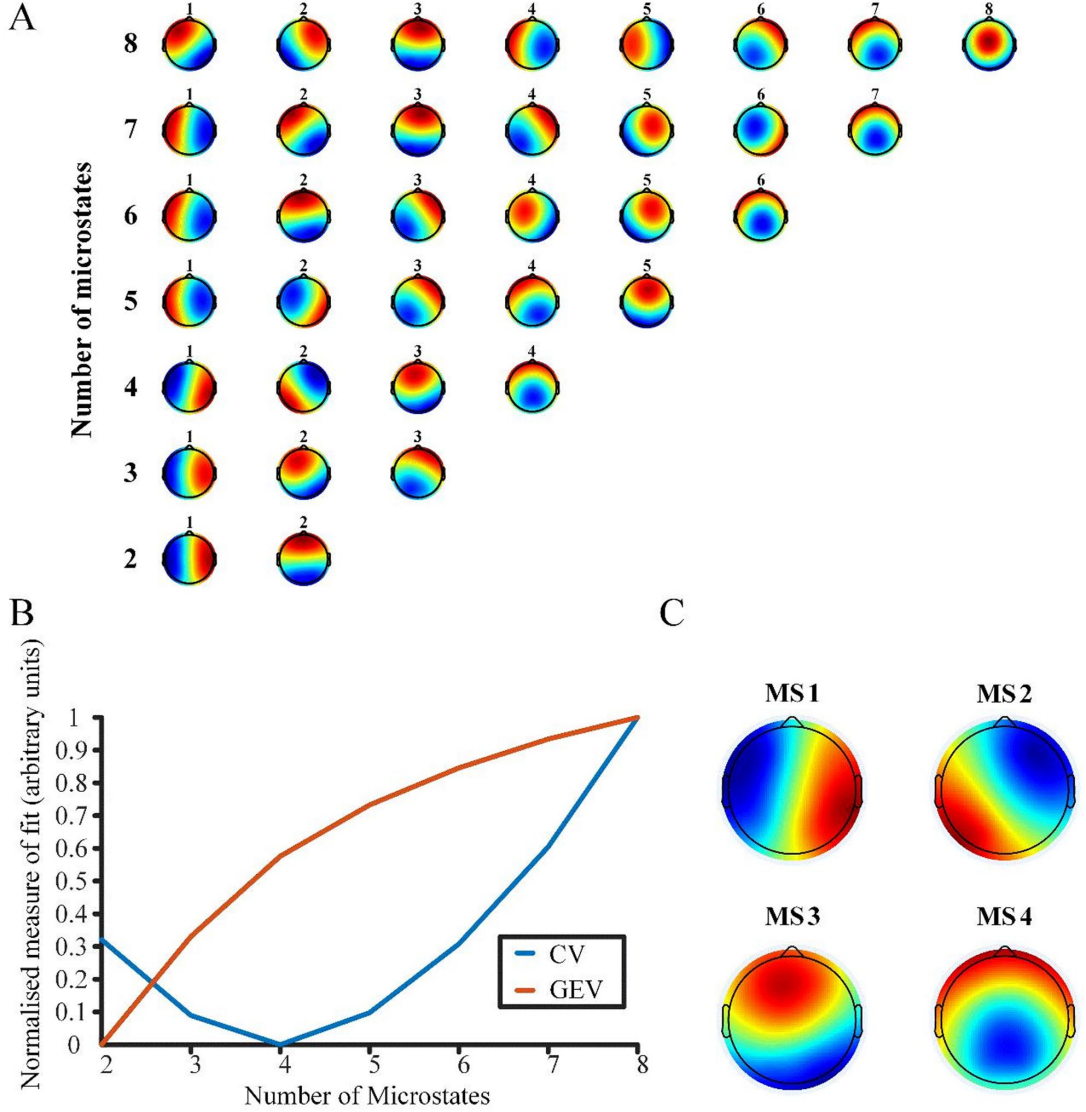
**A** Resting-state EEG microstates: scalp topographies for cluster solutions of 2 to 8 classes identified at GFP peaks. **B** Evaluation of Microstate Model Fit Using GEV and CV Criteria. **C** Spatial Topographies of the Four Selected Microstates

Figure 2A presents resting-state scalp topographies for microstate solutions containing two to eight classes, aggregated across all consciousness levels. The GFP series was obtained from 10-min EEG recordings, with peak detection constrained by a 10ms minimum inter-peak interval; 1,000 peaks were then randomly sampled per dataset. In the illustration, rows correspond to the chosen cluster counts, while columns (left to right) display the principal microstate prototypes.

To retain polarity independence, we quantified model fit with two complementary indices: GEV and CV. Using both metrics enhances analytical rigor and underpins the validity of the microstate model. Figure 2B illustrates how GEV and CV vary as the number of microstates rises. For straightforward comparison, each index was rescaled individually to the 0–1 interval.

Inspection of the fit curves indicated that models with three to five clusters were viable, and the four-cluster solution provided the optimal fit. We therefore selected four clusters for subsequent microstate analysis, balancing parsimony with explanatory strength. This configuration produced four canonical microstates (ms1–ms4) with distinct scalp topographies (Fig. 2C).

### Complexity of microstate sequences

**Fig. 3:**
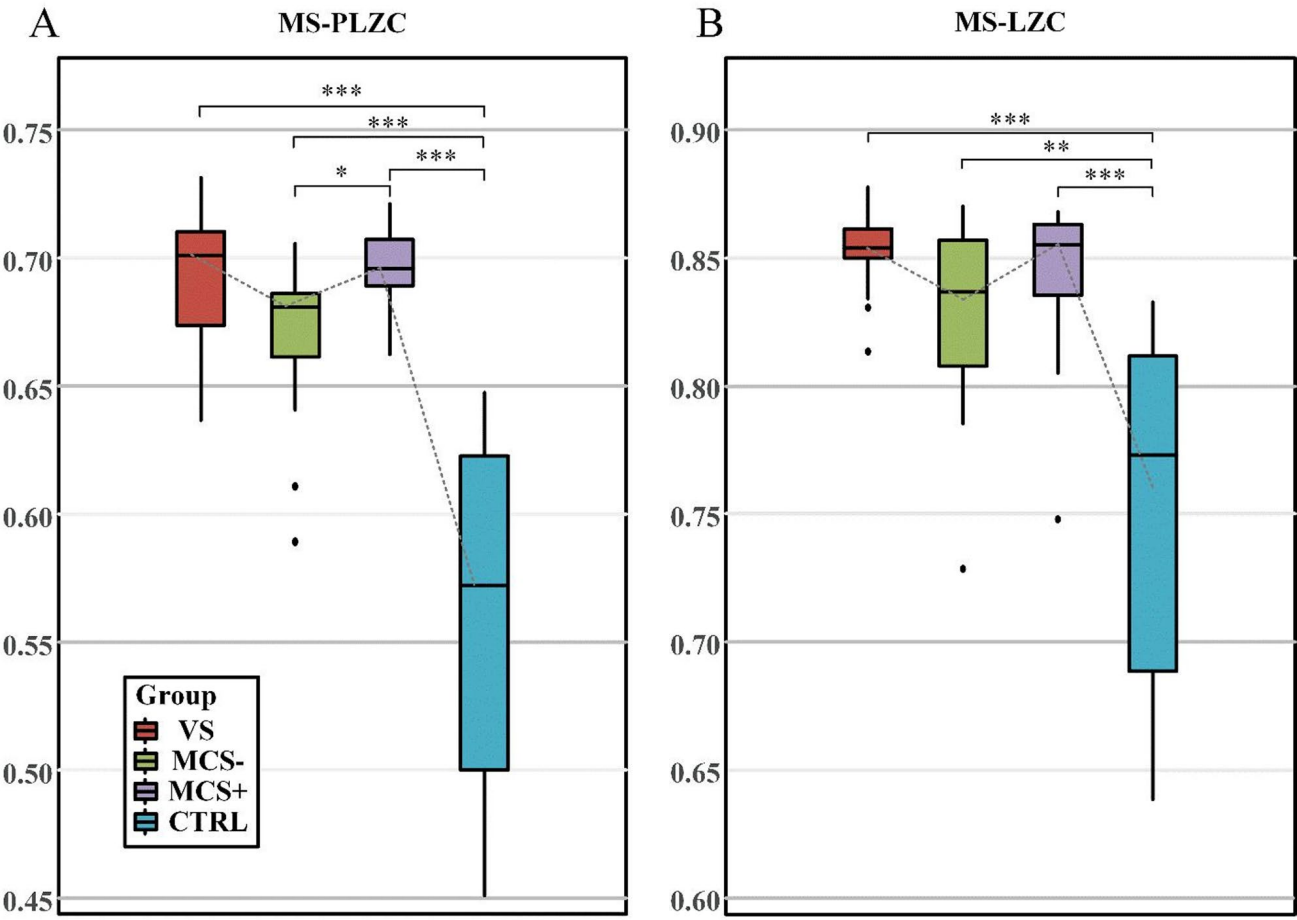
Presents two box plots—**A** for MS-PLZC and **B** for MS-LZC—depicting results from the four experimental groups (VS, MCS–, MCS+, CTRL). Each box plot displays the median, interquartile range, and outliers for each measure. Statistical significance is denoted as follows: $$\:*\:p\:\_adj\:<\:0.05,\:**\:p\:\_adj\:<\:0.01,\:and\:***\:p\:\_adj\:<\:0.001$$

Based on the Kruskal-Wallis test, statistically significant differences among the groups were observed for both the MS-LZC measure (H = 26.92, *p* < 0.0000, η²=0.2099) and the MS-PLZC measure (H = 35.11, *p* < 0.0000, η²=0.2816), with the MS-PLZC measure demonstrating greater statistical power. Furthermore, when comparing MCS + and MCS- using Cliff’s Delta, the effect size for the MS-LZC measure was − 0.3067 (indicating a medium effect; |d|<0.33), while the effect size for the MS-PLZC measure was − 0.6178 (indicating a large effect; |d|<0.474). This suggests that the MS-PLZC measure outperforms the MS-LZC measure in differentiating between levels of consciousness, especially when distinguishing MCS+ from MCS-.

In MS-PLZC (Fig. 3A), the CTRL group consistently displays lower complexity values compared to the other groups, showing a decrease in complexity with higher levels of consciousness, but exhibiting a turning point at MCS+. Specifically, the CTRL group’s permutation complexity is significantly lower than that of VS, MCS-, and MCS+ (p _adj < 0.001), while MCS- has significantly lower complexity than MCS+ (p _adj < 0.05) and lower (though not significantly) than VS. In MS-LZC (Fig. 3B), the complexity also decreases with increasing consciousness levels. The VS group exhibits higher median MS-LZC values than MCS- and CTRL, but no significant difference was found between VS and MCS-. MCS- shows significantly higher median MS-LZC values than CTRL, but lower than MCS+. Overall, no significant difference was observed between VS and MCS+. Notably, across both MS-PLZC and MS-LZC, the CTRL group shows markedly lower complexity than the other groups, especially when compared to VS.

### Relative power and temporal complexity

**Fig. 4:**
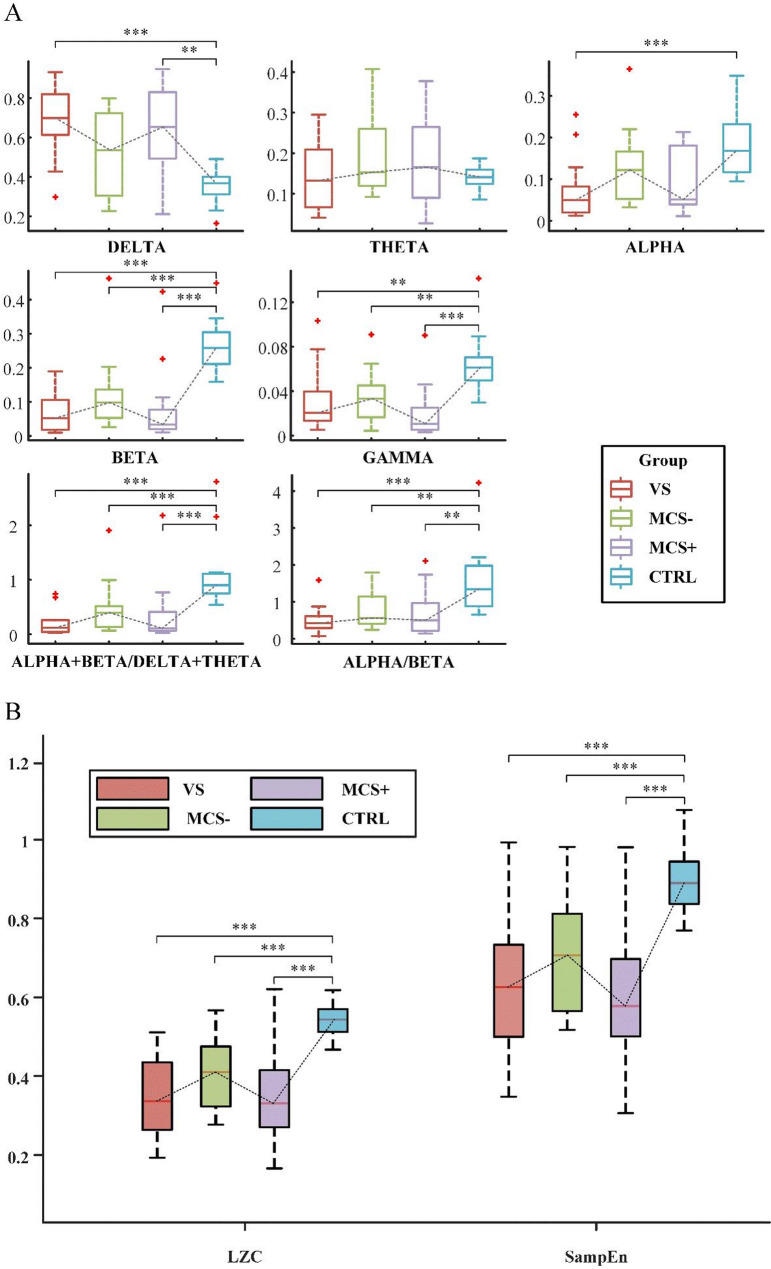
**A** Relative power and frequency band ratios across clinical groups. **B** Distribution of temporal complexity indices (LZC and SampEn) across clinical groups. Statistical significance is denoted as follows: $$\:*\:p\:\_adj\:<\:0.05,\:**\:p\:\_adj\:<\:0.01,\:and\:***\:p\:\_adj\:<\:0.001$$

In our study, we presented box plots (Fig. 4A) showing the distribution of relative power across EEG frequency bands (Delta, Theta, Alpha, Beta, Gamma) among different clinical groups (VS, MCS-, MCS+, CTRL), as well as power ratios of some frequency band combinations (Alpha+Beta/Delta+Theta, Alpha/Beta). Statistical analyses using Kruskal-Wallis tests and Cliff’s Delta yielded the following results:

Delta Band: *H* = 20.43, *p* = 0.0001, η² = 0.1529. Cliff’s Delta (MCS − vs. MCS+) = − 0.2622. The median relative power in the VS and MCS+ groups was significantly higher than that of the CTRL group. Theta Band: *H* = 2.95, *p* = 0.3988, η² = −0.0004. Cliff’s Delta (MCS − vs. MCS+) = 0.1556. Differences did not reach statistical significance. Alpha Band: *H* = 14.62, *p* = 0.0022, η² = 0.1019. Cliff’s Delta (MCS − vs. MCS+) = 0.1822. The relative power in the VS group was significantly lower than that of the CTRL group. Beta Band: *H* = 28.75, *p* < 0.0001, η² = 0.2259. Cliff’s Delta (MCS − vs. MCS+) = 0.4667. The median relative power in the VS, MCS-, and MCS+ groups was significantly lower than that of the CTRL group. Gamma Band: *H* = 21.50, *p* = 0.0001, η² = 0.1623. Cliff’s Delta (MCS − vs. MCS+) = 0.4489. Similar to Beta, the median relative power in the VS, MCS-, and MCS+ groups was significantly lower than that of the CTRL group. Alpha+Beta / Delta+Theta: *H* = 28.35, *p* < 0.0001, η² = 0.2223. Cliff’s Delta (MCS − vs. MCS+) = 0.3867. This ratio was lowest in the VS group and highest in the CTRL group, with the VS, MCS-, and MCS+ groups all significantly lower than the CTRL group. Alpha / Beta: *H* = 19.13, *p* = 0.0003, η² = 0.1415. Cliff’s Delta (MCS − vs. MCS+) = 0.1644. The ratio distribution showed a trend similar to Alpha+Beta/Delta+Theta, being significantly lower in VS, MCS-, and MCS+ compared to CTRL.

Notably, as the level of consciousness improved, the results of relative power exhibited a nonlinear change trend, with a turning point at the MCS+ group.

Figure 4B, we analyzed the distribution of two temporal complexity indices—Sample Entropy (SampEn) and Lempel-Ziv Complexity (LZC)—across different clinical groups (VS, MCS-, MCS+, CTRL). Statistical analyses using Kruskal-Wallis tests and Cliff’s Delta revealed the following:

SampEn: *H* = 23.94, *p* < 0.0001, η² = 0.1836, Cliff’s Delta (MCS − vs. MCS+) = 0.4222. The distribution of SampEn showed that the medians of the VS and MCS+ groups were significantly lower than that of the CTRL group. LZC: *H* = 27.88, *p* < 0.0001, η² = 0.2183, Cliff’s Delta (MCS − vs. MCS+) = 0.4044. The CTRL group’s values were significantly higher than those of the VS, MCS-, and MCS+ groups.

Similar to the results of relative power, the temporal complexity results also exhibited a nonlinear trend of change with increasing levels of consciousness, with a turning point at the MCS+ group (Table 2).

**Table 2:** Kruskal–Wallis test results (for VS, MCS−, MCS+, and CTRL) and Cliff’s Delta (MCS − vs. MCS+) for each frequency band, complexity measure, and microstate-based index (MS-LZC, MS-PLZC)

Feature	H	*p*	η²	Cliff’s Delta (MCS − vs. MCS+)
Delta	20.43	0.0001	0.1529	-0.2622
Theta	2.95	0.3988	-0.0004	0.1556
Alpha	14.62	0.0022	0.1019	0.1822
Beta	28.75	< 0.0000	0.2259	0.4667
Gamma	21.50	0.0001	0.1623	0.4489
(α + β)/(δ + θ)	28.35	< 0.0000	0.2223	0.3867
α/β	19.13	0.0003	0.1415	0.1644
LZC	27.88	< 0.0000	0.2183	0.4044
SampEn	23.94	< 0.0000	0.1836	0.4222
MS-LZC	26.92	< 0.0000	0.2099	-0.3067
**MS-PLZC**	**35.11**	**< 0.0000**	**0.2816**	**-0.6178**

In summary, building on MS-LZC, we proposed an improved MS-PLZC method and compared it with relative power, conventional temporal complexity measures, and MS-LZC itself. The results showed that MS-PLZC exhibited stronger discriminative power in distinguishing levels of consciousness, particularly in differentiating MCS− from MCS+, where its effect size (Cliff’s Delta = − 0.6178) was significantly greater than that of MS-LZC (− 0.3067), and also outperformed relative power and conventional temporal complexity measures.

**Table 3:** Classification results of each feature in LOOCV

Feature	Acc	Sen	Spe	AUC
Delta	0.433	0.467	0.4	0.524
Theta	0.7	**0.8**	0.6	0.716
Alpha	0.633	0.733	0.533	0.516
Beta	0.533	0.667	0.4	0.618
Gamma	0.567	0.6	0.533	0.542
(α + β)/(δ + θ)	0.533	0.6	0.467	0.6
α/β	0.5	0.533	0.467	0.551
Power-7	0.333	0.333	0.333	0.711
LZC	0.6	0.533	0.667	0.578
SampEn	0.5	0.4	0.6	0.516
NL-2	0.6	0.6	0.6	0.6
MS-LZC	0.533	0.733	0.333	0.591
**MS-PLZC**	**0.733**	0.733	**0.733**	**0.733**

**Fig. 5:**
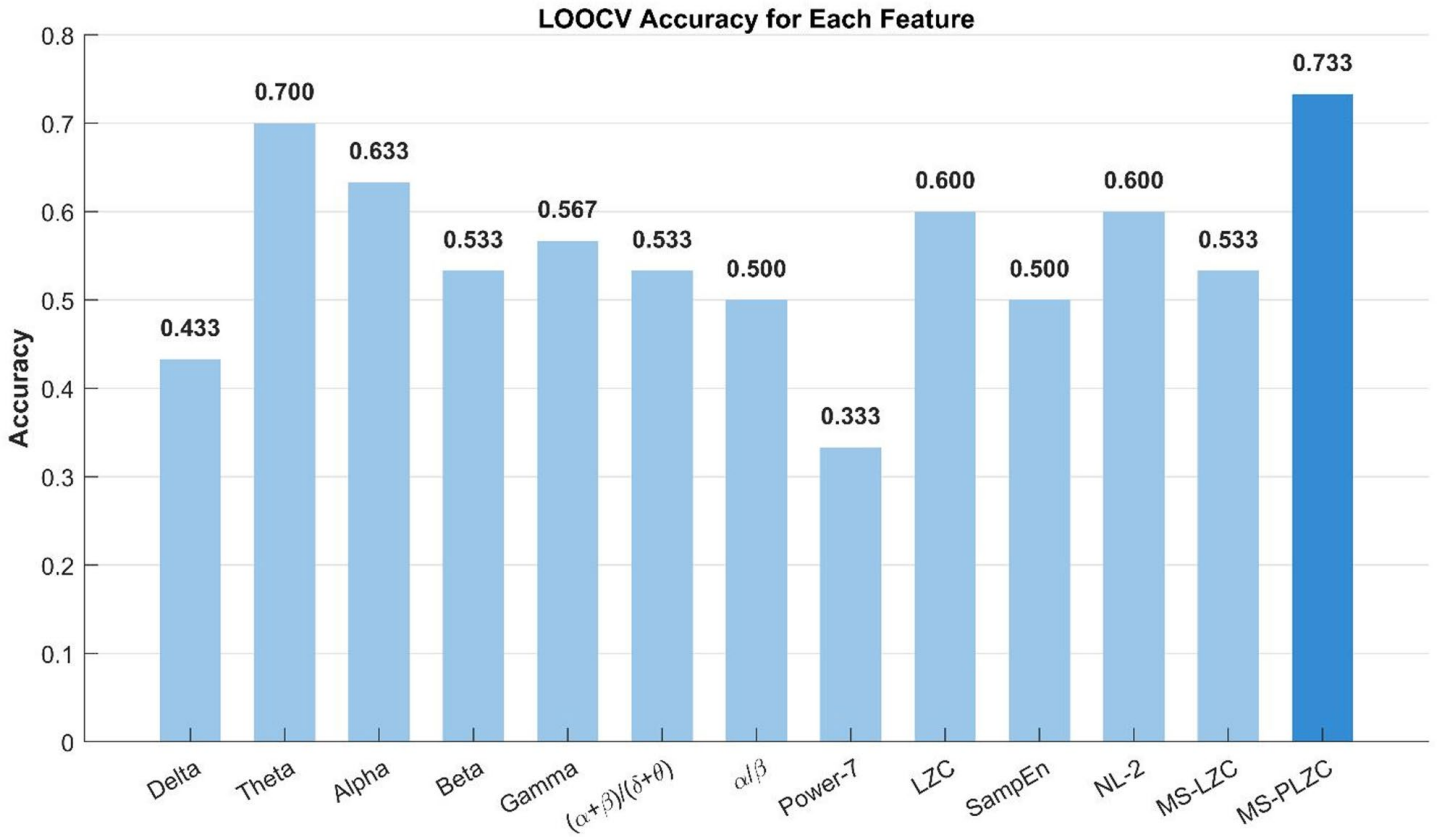
Bar chart of LOOCV accuracies (the dark bar highlights the best-performing feature, MS-PLZC)

In the LOOCV evaluation of 30 participants, the classification performance of each feature is summarized in Table 3 and illustrated in Fig. 5. Considerable variability was observed among single power-band features: the Θ band yielded the highest accuracy (Acc = 0.700) and, across all features, the highest sensitivity (Sen = 0.800, Spe = 0.600, AUC = 0.716), whereas the Δ band and the Power-7 combination produced the lowest accuracies (Acc = 0.433 and 0.333, respectively). Among individual nonlinear metrics, LZC achieved an accuracy of 0.600 and the highest specificity (Spe = 0.667); SampEn exhibited an AUC comparable to the power features but a relatively low sensitivity (0.400). Combining LZC and SampEn (NL-2) balanced sensitivity and specificity (both 0.600) yet did not further improve overall accuracy.

Features derived from EEG microstates outperformed conventional power and nonlinear indices. Although MS-LZC increased sensitivity to 0.733, its specificity dropped to 0.333, limiting its overall effectiveness. By contrast, the proposed MS-PLZC achieved the highest accuracy (0.733), specificity (0.733), and AUC (0.733), while attaining a sensitivity of 0.733—second only to the $\:\theta\:$ band—thus delivering the best overall performance. Compared with the best traditional single feature ($\:\theta\:$ band), MS-PLZC improved accuracy by 3.3% points and exceeded the Power-7 combination by 40% points. The bar chart likewise shows that, aside from MS-PLZC, only the $\:\theta\:$ band reaches or surpasses an accuracy of 0.700.

Overall, MS-PLZC markedly enhances the separability between MCS + and MCS- without increasing feature dimensionality, providing a new and effective discriminator for EEG-based assessment of consciousness.

## Discussion

In this study, we conducted a systematic EEG microstate analysis in DOC patients with different levels of consciousness and compared the existing microstate complexity measure (MS-LZC), relative power, and conventional temporal complexity indices with our newly proposed MS-PLZC. The results show that MS-PLZC excels at capturing the subtle differences between MCS − and MCS+, demonstrating the strongest discriminative power and revealing a nonlinear turning point at the MCS+ stage. The following discussion will further elaborate on the impact of these results on understanding the brain function of DOC patients and their potential clinical application value.

### From disorder to order: how EEG microstate complexity reveals the critical turning point in consciousness evolution

In recent years, EEG microstate analysis has emerged as a powerful tool for examining the temporal dynamics of whole-brain networks and may serve as a non-invasive functional biomarker for neurological disorders [28, 36–42]. Analyzing the transition probabilities and complexity of microstate sequences characterizes brain activity under different conditions [43] and offers insight into states of consciousness [44, 45].

In this study, we applied two metrics—MS-LZC and MS-PLZC—to four groups (VS, MCS–, MCS+, CTRL). Both measures revealed a relationship between the level of consciousness and microstate complexity. MS-LZC, reflecting the randomness of data, showed higher values in lower consciousness states (VS); however, this relationship was not strictly monotonic across all DoC subgroups, as MCS+ showed numerically higher MS-LZC than MCS − in our cohort. MS-LZC indicating more irregular EEG activity, and decreased as consciousness recovered, suggesting a shift toward more ordered patterns [46, 47].

MS-PLZC, focusing on permutations within EEG sequences, similarly exhibited higher values in VS and significantly lower values in CTRL. Notably, as consciousness improves, MS-PLZC values decrease—indicating more orderly, less random brain activity—yet an inflection point in the MCS+ group marks a transitional equilibrium in microstate dynamics [48].

Spatiotemporal complexity (including microstate permutation complexity and Lempel-Ziv complexity) reflects how EEG activity is organized across space and time. At lower consciousness levels, weak functional connectivity leads to more disordered and random EEG patterns, manifesting as higher spatiotemporal complexity. As consciousness improves, connectivity strengthens and EEG dynamics become more coordinated, reducing overall complexity and highlighting a transition from disorder to order and from inefficiency to efficiency [49, 50].

### Inflection point

Wilkinson et al. found significant nonlinear EEG changes with age in children, suggesting that the observed inflection point reflects partial functional recovery and cortico-thalamic network reorganization [51]. Liang also identified substantial nonlinear EEG dynamics between ages 3–6, linking this critical period to key stages of brain network development [52]. Drawing on these developmental findings only as a cautious parallel, we observed non-linear shifts in EEG relative power, temporal complexity, and microstate complexity among patients with disorders of consciousness, with an apparent inflection at the MCS+ group. Importantly, our updated demographic analysis showed that aetiology distributions differed across the patient groups (TBI/stroke/hypoxia, *P* = 0.010), with the MCS+ group being enriched for traumatic brain injury and relatively under-represented for hypoxia. Therefore, the observed non-monotonic pattern may reflect a combination of recovery-stage effects and aetiology-related heterogeneity, and should be interpreted as exploratory rather than a definitive mechanistic signature. Accordingly, we hypothesize that MCS + may represent a transitional stage of consciousness recovery, potentially characterized by partial network reorganization and a shift toward greater dynamic adaptability; however, this interpretation warrants further validation in larger, aetiology-matched or stratified cohorts.

We further note that MS-PLZC effectively distinguishes MCS+ from MCS − in our cohort, suggesting that microstate-sequence organization changes meaningfully at this boundary. We speculate that such changes may relate to partial recovery and reorganization of cortico-thalamic and distributed cortical networks [53], which could increase state-transition complexity and contribute to the observed inflection; however, given the between-group differences in aetiology composition, this explanation should be considered tentative and requires confirmation under designs that control for aetiology (e.g., stratification/matching or covariate-adjusted models).

During this period, patients may regain basic self-awareness, follow simple commands, and communicate functionally. Those who surpass MCS+ often progress to higher consciousness levels (e.g., exiting the minimally conscious state, eMCS) and thus no longer meet criteria for disorders of consciousness. Consistent with this clinical framework, future studies should examine whether the non-linear pattern persists when controlling for clinical covariates (including aetiology and time since injury) and whether it generalizes across external cohorts.

### Limitations and future research directions

Despite the encouraging outcomes of this study, several limitations should be noted. First, the relatively small sample size constrains the generalizability of our findings, and the cross-sectional design makes it difficult to dynamically track changes in EEG markers over time. Nevertheless, the cohort size in the present work is relatively larger than that of many prior EEG studies in disorders of consciousness, given the practical constraints of clinical recruitment and bedside data quality control. Second, reliance on specific analytical methods (e.g., LZC and our proposed MS-PLZC) may introduce potential biases or overlook other neurologically relevant features. In task-driven EEG classification (e.g., motor/mental imagery BCI), automated pipelines that integrate nonstationary decomposition, multi-domain feature fusion, and learning-based classifiers have shown strong performance and provide useful benchmarking references; however, our study targets biomarker discovery in clinically constrained DoC cohorts, where interpretability and robustness under small, heterogeneous bedside datasets are prioritized, and thus such large-scale decomposition/deep-learning baselines were not included in the current scope [54–56]. Similarly, classical decomposition/time–frequency approaches (e.g., EWT- or VMD/MVMD-based frameworks) can provide explicit mode separation and finer time–frequency resolution, but they often introduce additional parameter choices and computational cost; therefore, we did not include them as primary baselines in this study focused on bedside DoC EEG [57–64]. Relatedly, advanced denoising or decomposition-driven preprocessing (e.g., MSPCA, wavelet/EWT-based denoising) may benefit rhythm-specific and non-linear descriptor extraction, but would require systematic ablations and stability tests to isolate the impact of denoising choices from downstream modeling under limited and heterogeneous DoC data [57, 58, 65–68]. For instance, MS-PLZC requires the predefined number of microstates (fixed to four in this study), which might be insufficient to capture subtler EEG patterns in larger or more heterogeneous samples. In addition, phase-space/recurrence- or geometry-based dynamical descriptors (e.g., Poincaré-plot or reconstructed phase-space features) may offer complementary information at the local signal dynamics level, and future work could benchmark and/or fuse them with microstate-sequence complexity to assess complementarity under DoC settings [66, 67, 69, 70].

In addition, the MCS+ subgroup often exhibits fluctuating levels of consciousness, which affects the stability of EEG measurements and complicates comparisons across different time points. The unexpectedly low α power observed in MCS+ also suggests that this subgroup may represent a distinct and diverse stage in the consciousness recovery process. It may reflect subgroup heterogeneity and/or clinical confounders (e.g., differences in aetiology composition and arousal), rather than a definitive stage marker.

Future research should incorporate larger samples and adopt longitudinal or repeated-measures designs to more comprehensively map dynamic changes in consciousness, accurately evaluate rehabilitation interventions, and test whether the observed “inflection point” persists within individuals over time after controlling for clinically relevant covariates (e.g., aetiology, time since injury, and arousal-related CRS-R subscores). Integrating EEG with other neuroimaging approaches, such as functional magnetic resonance imaging and positron emission tomography (fMRI, PET), may further elucidate the underlying neural mechanisms of DOC. Moreover, leveraging machine learning and more advanced signal processing techniques (e.g., topology-aware representations, transfer learning on time–frequency images, and 1D CNN/LSTM-type temporal models), along with the fusion of MS-PLZC features and traditional metrics like relative power and temporal complexity, could significantly enhance the diagnostic accuracy and robustness of EEG-based measures. Such extensions would be most informative when accompanied by per-class metrics, calibration-oriented analyses, robustness/sensitivity tests to noise and artifacts, and—where feasible—external dataset validation [54–56, 71–73]. Lastly, continuously refining and validating MS-PLZC under varying parameter settings and patient populations is crucial for its broader clinical application.

## Conclusions

In this study, we proposed and validated a novel EEG microstate-based spatiotemporal complexity metric, termed MS-PLZC, and compared it against relative power, conventional temporal complexity indices, as well as the existing MS-LZC method in patients with disorders of consciousness. Our results indicate that MS-PLZC exhibits a stronger ability to differentiate MCS− from MCS+, particularly by capturing a distinct nonlinear turning point at the MCS+ stage. These findings underscore its potential for more precise evaluation of consciousness states and highlight its promise as a pivotal tool for identifying, monitoring, and intervening in subtle shifts in consciousness.

## Supplementary Information

Supplementary Material 1.

## Data Availability

The data that support the findings of this study are available on reasonable request from the corresponding author.

## Abbreviations

DOC: Disorders of consciousness
EEG: Electroencephalography
UWS: Unresponsive wakefulness syndrome
VS: Vegetative state
MCS: Minimally conscious state
CTRL: Healthy control
LZC: Lempel–Ziv complexity
PLZC: Permutation Lempel–Ziv complexity
MS-LZC: Microstate Lempel–Ziv complexity
MS-PLZC: Microstate permutation Lempel–Ziv complexity
LOOCV: Leave-one-out cross-validation
AUC: Area under the curve
ICA: Independent component analysis
CRS-R: Coma recovery scale-revised
GEV: Global explained variance
CV: Cross-validation
GFP: Global field power
qEEG: Quantitative EEG

## References

[1] Giacino JT, Fins JJ, Laureys S, Schiff ND. Disorders of consciousness after acquired brain injury: the state of the science. Nat Rev Neurol. 2014;10(2):99–114. 24468878 10.1038/nrneurol.2013.279

[2] Schiff ND, Plum F. The role of arousal and gating systems in the neurology of impaired consciousness. J Clin Neurophysiol. 2000;17(5):438–52. 11085547 10.1097/00004691-200009000-00002

[3] Jiang T. Recent Progress in Basic and Clinical Research on Disorders of Consciousness. Neurosci Bull. 2018;34(4):589–91. 30039245 10.1007/s12264-018-0264-0 PMC6060211

[4] Marsden C. The Diagnosis of Stupor and Coma. J Neurol Neurosurg Psychiatry. 1981;44(3):270–1. 3rd ed.

[5] Laureys S, Celesia GG, Cohadon F, Lavrijsen J, León-Carrión J, Sannita WG, Sazbon L, Schmutzhard E, von Wild KR, Zeman A, et al. Unresponsive wakefulness syndrome: a new name for the vegetative state or apallic syndrome. BMC Med. 2010;8:68. 21040571 10.1186/1741-7015-8-68 PMC2987895

[6] Giacino JT, Ashwal S, Childs N, Cranford R, Jennett B, Katz DI, Kelly JP, Rosenberg JH, Whyte J, Zafonte RD, et al. The minimally conscious state: definition and diagnostic criteria. Neurology. 2002;58(3):349–53. 11839831 10.1212/wnl.58.3.349

[7] Bruno M-A, Vanhaudenhuyse A, Thibaut A, Moonen G, Laureys S. From unresponsive wakefulness to minimally conscious PLUS and functional locked-in syndromes: recent advances in our understanding of disorders of consciousness. J Neurol. 2011;258:1373–84. 21674197 10.1007/s00415-011-6114-x

[8] Bruno MA, Vanhaudenhuyse A, Thibaut A, Moonen G, Laureys S. From unresponsive wakefulness to minimally conscious PLUS and functional locked-in syndromes: recent advances in our understanding of disorders of consciousness. J Neurol. 2011;258(7):1373–84. 21674197 10.1007/s00415-011-6114-x

[9] Giacino JT, Kalmar K, Whyte J. The JFK Coma Recovery Scale-Revised: measurement characteristics and diagnostic utility. Arch Phys Med Rehabil. 2004;85(12):2020–9. 15605342 10.1016/j.apmr.2004.02.033

[10] Luauté J, Maucort-Boulch D, Tell L, Quelard F, Sarraf T, Iwaz J, Boisson D, Fischer C. Long-term outcomes of chronic minimally conscious and vegetative states. Neurology. 2010;75(3):246–52. 20554940 10.1212/WNL.0b013e3181e8e8df

[11] Schnakers C, Vanhaudenhuyse A, Giacino J, Ventura M, Boly M, Majerus S, Moonen G, Laureys S. Diagnostic accuracy of the vegetative and minimally conscious state: Clinical consensus versus standardized neurobehavioral assessment. BMC Neurol. 2009;9(1):35. 19622138 10.1186/1471-2377-9-35 PMC2718857

[12] Nuwer M. Assessment of digital EEG, quantitative EEG, and EEG brain mapping: report of the American Academy of Neurology and the American Clinical Neurophysiology Society. Neurology. 1997;49(1):277–92. 9222209 10.1212/wnl.49.1.277

[13] Zhang H, Zhou QQ, Chen H, Hu XQ, Li WG, Bai Y, Han JX, Wang Y, Liang ZH, Chen D, et al. The applied principles of EEG analysis methods in neuroscience and clinical neurology. Mil Med Res. 2023;10(1):67. 38115158 10.1186/s40779-023-00502-7 PMC10729551

[14] Xin X, Gao Y, Zhang H, Cao K, Shi Y. Correlation of continuous electroencephalogram with clinical assessment scores in acute stroke patients. Neurosci Bull. 2012;28(5):611–7. 22965744 10.1007/s12264-012-1265-z PMC5561917

[15] Chen P, Xie Q, Wu X, Huang H, Lv W, Chen L, Guo Y, Zhang S, Hu H, Wang Y, et al. Abnormal Effective Connectivity of the Anterior Forebrain Regions in Disorders of Consciousness. Neurosci Bull. 2018;34(4):647–58. 29959668 10.1007/s12264-018-0250-6 PMC6060215

[16] Xu C, Yuan Z, Chen Z, Liao Z, Li S, Feng Y, Tang Z, Nian J, Huang X, Zhong H, et al. Perturbational complexity index in assessing responsiveness to rTMS treatment in patients with disorders of consciousness: a cross-over randomized controlled trial study. J Neuroeng Rehabil. 2024;21(1):167. 39300529 10.1186/s12984-024-01455-1 PMC11411826

[17] Stender J, Gosseries O, Bruno M-A, Charland-Verville V, Vanhaudenhuyse A, Demertzi A, Chatelle C, Thonnard M, Thibaut A, Heine L, et al. Diagnostic precision of PET imaging and functional MRI in disorders of consciousness: a clinical validation study. Lancet. 2014;384(9942):514–22. 24746174 10.1016/S0140-6736(14)60042-8

[18] Sinitsyn DO, Poydasheva AG, Bakulin IS, Legostaeva LA, Iazeva EG, Sergeev DV, Sergeeva AN, Kremneva EI, Morozova SN, Lagoda DY et al. Detecting the Potential for Consciousness in Unresponsive Patients Using the Perturbational Complexity Index. Brain Sci 2020, 10(12). 10.3390/brainsci10120917 PMC7760168 33260944

[19] Engemann DA, Raimondo F, King J-R, Rohaut B, Louppe G, Faugeras F, Annen J, Cassol H, Gosseries O, Fernandez-Slezak D, et al. Robust EEG-based cross-site and cross-protocol classification of states of consciousness. Brain. 2018;141(11):3179–92. 30285102 10.1093/brain/awy251

[20] Hermann B, Stender J, Habert MO, Kas A, Denis-Valente M, Raimondo F, Pérez P, Rohaut B, Sitt JD, Naccache L. Multimodal FDG-PET and EEG assessment improves diagnosis and prognostication of disorders of consciousness. Neuroimage Clin. 2021;30:102601. 33652375 10.1016/j.nicl.2021.102601 PMC7921007

[21] Lv S, Ran X, Xia M, Zhang Y, Pang T, Zhou X, Zhao Z, Yu Y, Gao Z. Classification of left and right-hand motor imagery in acute stroke patients using EEG microstate. J Neuroeng Rehabil. 2025;22(1):137. 40533772 10.1186/s12984-025-01668-y PMC12178014

[22] Lehmann D, Ozaki H, Pal I. EEG alpha map series: brain micro-states by space-oriented adaptive segmentation. Electroencephalogr Clin Neurophysiol. 1987;67(3):271–88. 2441961 10.1016/0013-4694(87)90025-3

[23] Pascual-Marqui RD, Michel CM, Lehmann D. Segmentation of brain electrical activity into microstates: model estimation and validation. IEEE Trans Biomed Eng. 1995;42(7):658–65. 7622149 10.1109/10.391164

[24] Koenig T, Prichep L, Lehmann D, Sosa PV, Braeker E, Kleinlogel H, Isenhart R, John ER. Millisecond by Millisecond, Year by Year: Normative EEG Microstates and Developmental Stages. NeuroImage. 2002;16(1):41–8. 11969316 10.1006/nimg.2002.1070

[25] Lehmann D, Strik WK, Henggeler B, Koenig T, Koukkou M. Brain electric microstates and momentary conscious mind states as building blocks of spontaneous thinking: I. Visual imagery and abstract thoughts. Int J Psychophysiol. 1998;29(1):1–11. 9641243 10.1016/s0167-8760(97)00098-6

[26] Lempel A, Ziv J. On the complexity of finite sequences. IEEE Trans Inf Theory. 1976;22(1):75–81.

[27] Li D, Fabus MS, Sleigh JW. Brain Complexities and Anesthesia: Their Meaning and Measurement. Anesthesiology. 2022;137(3):290–302. 35925575 10.1097/ALN.0000000000004293

[28] Tait L, Tamagnini F, Stothart G, Barvas E, Monaldini C, Frusciante R, Volpini M, Guttmann S, Coulthard E, Brown JT, et al. EEG microstate complexity for aiding early diagnosis of Alzheimer’s disease. Sci Rep. 2020;10(1):17627. 33077823 10.1038/s41598-020-74790-7 PMC7572485

[29] Yu F, Gao Y, Li F, Zhang X, Hu F, Jia W, Li X. Resting-state EEG microstates as electrophysiological biomarkers in post-stroke disorder of consciousness. Front Neurosci. 2023;17:1257511. 37849891 10.3389/fnins.2023.1257511 PMC10577186

[30] Teng C, Cong L, Tian Q, Liu K, Cheng S, Zhang T, Dang W, Hou Y, Ma J, Hui D, et al. EEG microstate in people with different degrees of fear of heights during virtual high-altitude exposure. Brain Res Bull. 2024;218:111112. 39486463 10.1016/j.brainresbull.2024.111112

[31] Yin J, Sui W, Zhuang X, Sheng Y, Li Y. An Improved Lempel–Ziv Complexity for Bearing Fault Diagnosis Based on the Time–Frequency Encoding Method. IEEE Trans Instrum Meas. 2025;74:1–12.42146727

[32] Noman K, Ali U, Li Y, Wang S, Patwari AU, Kumar A. A Novel Nonlinear Dynamic Measure for Early Detection of Bearing Fault Using Weighted Squared Envelope-Based Symbolic Lempel-Ziv Complexity. IEEE Trans Instrum Meas. 2024;73:1–16.

[33] Li Y, Cheng X, Wu J, Yan Y. Multivariate Variable-Step Multiscale Extended Dispersion Entropy-Based Lempel–Ziv Complexity and Its Application in Fault Diagnosis. IEEE Trans Instrum Meas. 2025;74:1–12.42146727

[34] Olofsen E, Sleigh JW, Dahan A. Permutation entropy of the electroencephalogram: a measure of anaesthetic drug effect. Br J Anaesth. 2008;101(6):810–21. 18852113 10.1093/bja/aen290

[35] Hu J, Gao J, Principe JC. Analysis of biomedical signals by the Lempel-Ziv complexity: the effect of finite data size. IEEE Trans Biomed Eng. 2006;53(12):2606–9. 17152441 10.1109/TBME.2006.883825

[36] da Cruz JR, Favrod O, Roinishvili M, Chkonia E, Brand A, Mohr C, Figueiredo P, Herzog MH. EEG microstates are a candidate endophenotype for schizophrenia. Nat Commun. 2020;11(1):3089. 32555168 10.1038/s41467-020-16914-1 PMC7303216

[37] Klepl D, He F, Wu M, Marco M, Blackburn DJ, Sarrigiannis PG. Characterising Alzheimer’s Disease With EEG-Based Energy Landscape Analysis. IEEE J Biomed Health Inf. 2022;26(3):992–1000. 10.1109/JBHI.2021.3105397 34406951

[38] Michel CM, Koenig T, Neuroimage. 2018, 180(Pt B):577–93. 10.1016/j.neuroimage.2017.11.062 29196270

[39] Milz P, Pascual-Marqui RD, Achermann P, Kochi K, Faber PL. The EEG microstate topography is predominantly determined by intracortical sources in the alpha band. NeuroImage. 2017;162:353–61. 28847493 10.1016/j.neuroimage.2017.08.058

[40] Musaeus CS, Nielsen MS, Høgh P. Microstates as Disease and Progression Markers in Patients With Mild Cognitive Impairment. Front Neurosci. 2019;13:563. 31263397 10.3389/fnins.2019.00563 PMC6584800

[41] Nishida K, Morishima Y, Yoshimura M, Isotani T, Irisawa S, Jann K, Dierks T, Strik W, Kinoshita T, Koenig T. EEG microstates associated with salience and frontoparietal networks in frontotemporal dementia, schizophrenia and Alzheimer’s disease. Clin Neurophysiol. 2013;124(6):1106–14. 23403263 10.1016/j.clinph.2013.01.005

[42] van der Zande JJ, Gouw AA, van Steenoven I, Scheltens P, Stam CJ, Lemstra AW. EEG Characteristics of Dementia With Lewy Bodies, Alzheimer’s Disease and Mixed Pathology. Front Aging Neurosci. 2018;10:190. 30018548 10.3389/fnagi.2018.00190 PMC6037893

[43] Gschwind M, Michel CM, Van De Ville D. Long-range dependencies make the difference-Comment on A stochastic model for EEG microstate sequence analysis. NeuroImage. 2015;117:449–55. 26032884 10.1016/j.neuroimage.2015.05.062

[44] Bréchet L, Brunet D, Birot G, Gruetter R, Michel CM, Jorge J. Capturing the spatiotemporal dynamics of self-generated, task-initiated thoughts with EEG and fMRI. NeuroImage. 2019;194:82–92. 30902640 10.1016/j.neuroimage.2019.03.029

[45] Lehmann D, Faber PL, Galderisi S, Herrmann WM, Kinoshita T, Koukkou M, Mucci A, Pascual-Marqui RD, Saito N, Wackermann J, et al. EEG microstate duration and syntax in acute, medication-naive, first-episode schizophrenia: a multi-center study. Psychiatry Res. 2005;138(2):141–56. 15766637 10.1016/j.pscychresns.2004.05.007

[46] Bréchet L, Michel CM. EEG Microstates in Altered States of Consciousness. Front Psychol. 2022;13:856697. 35572333 10.3389/fpsyg.2022.856697 PMC9094618

[47] Artoni F, Maillard J, Britz J, Seeber M, Lysakowski C, Bréchet L, Tramèr MR, Michel CM. EEG microstate dynamics indicate a U-shaped path to propofol-induced loss of consciousness. NeuroImage. 2022;256:119156. 35364276 10.1016/j.neuroimage.2022.119156

[48] Thibaut A, Bodien YG, Laureys S, Giacino JT. Minimally conscious state plus: diagnostic criteria and relation to functional recovery. J Neurol. 2020;267(5):1245–54. 31773246 10.1007/s00415-019-09628-y

[49] Bai Y, Xia X, Li X. A Review of Resting-State Electroencephalography Analysis in Disorders of Consciousness. Front Neurol. 2017;8:471. 28955295 10.3389/fneur.2017.00471 PMC5601979

[50] Varley TF, Craig M, Adapa R, Finoia P, Williams G, Allanson J, Pickard J, Menon DK, Stamatakis EA. Fractal dimension of cortical functional connectivity networks & severity of disorders of consciousness. PLoS ONE. 2020;15(2):e0223812. 32053587 10.1371/journal.pone.0223812 PMC7017993

[51] Wilkinson CL, Yankowitz LD, Chao JY, Gutiérrez R, Rhoades JL, Shinnar S, Purdon PL, Nelson CA. Developmental trajectories of EEG aperiodic and periodic components in children 2–44 months of age. Nat Commun. 2024;15(1):5788. 38987558 10.1038/s41467-024-50204-4 PMC11237135

[52] Liang Z, Ren N, Wen X, Li H, Guo H, Ma Y, Li Z, Li X. Age-dependent cross frequency coupling features from children to adults during general anesthesia. NeuroImage. 2021;240:118372. 34245867 10.1016/j.neuroimage.2021.118372

[53] Stitt I, Hollensteiner KJ, Galindo-Leon E, Pieper F, Fiedler E, Stieglitz T, Engler G, Nolte G, Engel AK. Dynamic reconfiguration of cortical functional connectivity across brain states. Sci Rep. 2017;7(1):8797. 28821753 10.1038/s41598-017-08050-6 PMC5562766

[54] Sadiq MT, Yu X, Yuan Z, Aziz MZ, Siuly S, Ding W. Toward the Development of Versatile Brain–Computer Interfaces. IEEE Trans Artif Intell. 2021;2(4):314–28.

[55] Yu X, Aziz MZ, Sadiq MT, Fan Z, Xiao G. A New Framework for Automatic Detection of Motor and Mental Imagery EEG Signals for Robust BCI Systems. IEEE Trans Instrum Meas. 2021;70:1–12.33776080

[56] Yu X, Aziz MZ, Sadiq MT, Jia K, Fan Z, Xiao G. Computerized Multidomain EEG Classification System: A New Paradigm. IEEE J Biomedical Health Inf. 2022;26(8):3626–37. 10.1109/JBHI.2022.3151570 35157605

[57] Akbari H, Sadiq MT. Detection of focal and non-focal EEG signals using non-linear features derived from empirical wavelet transform rhythms. Phys Eng Sci Med. 2021;44(1):157–71. 33417158 10.1007/s13246-020-00963-3

[58] Akbari H, Sadiq MT, Rehman AU. Classification of normal and depressed EEG signals based on centered correntropy of rhythms in empirical wavelet transform domain. Health Inform Sci Syst. 2021;9(1):9. 10.1007/s13755-021-00139-7 PMC7867675 33604030

[59] Sadiq MT, Yu X, Yuan Z, Aziz MZ. Motor imagery BCI classification based on novel two-dimensional modelling in empirical wavelet transform. Electron Lett. 2020;56(25):1367–9.

[60] Sadiq MT, Yu X, Yuan Z, Aziz MZ. Identification of Motor and Mental Imagery EEG in Two and Multiclass Subject-Dependent Tasks Using Successive Decomposition Index. In: Sensors. vol. 20; 2020: 5283. 10.3390/s20185283 PMC7570740 32947766

[61] Sadiq MT, Yu X, Yuan Z, Aziz MZ, Rehman Nu, Ding W, Xiao G. Motor Imagery BCI Classification Based on Multivariate Variational Mode Decomposition. IEEE Trans Emerg Top Comput Intell. 2022;6(5):1177–89.

[62] Sadiq MT, Yu X, Yuan Z, Aziz MZ, Siuly S, Ding W. A Matrix Determinant Feature Extraction Approach for Decoding Motor and Mental Imagery EEG in Subject-Specific Tasks. IEEE Trans Cogn Dev Syst. 2022;14(2):375–87.

[63] Sadiq MT, Yu X, Yuan Z, Fan Z, Rehman AU, Li G, Xiao G. Motor Imagery EEG Signals Classification Based on Mode Amplitude and Frequency Components Using Empirical Wavelet Transform. IEEE Access. 2019;7:127678–92.

[64] Sadiq MT, Yu X, Yuan Z, Zeming F, Rehman AU, Ullah I, Li G, Xiao G. Motor Imagery EEG Signals Decoding by Multivariate Empirical Wavelet Transform-Based Framework for Robust Brain–Computer Interfaces. IEEE Access. 2019;7:171431–51.

[65] Sadiq MT, Akbari H, Siuly S, Yousaf A, Rehman AU. A novel computer-aided diagnosis framework for EEG-based identification of neural diseases. Comput Biol Med. 2021;138:104922. 34656865 10.1016/j.compbiomed.2021.104922

[66] Sadiq MT, Akbari H, Siuly S, Li Y, Wen P. Alcoholic EEG signals recognition based on phase space dynamic and geometrical features. Chaos Solitons Fractals. 2022;158:112036.

[67] Sadiq MT, Yousaf A, Siuly S, Almogren A. Fast Fractional Fourier Transform-Aided Novel Graphical Approach for EEG Alcoholism Detection. In: Bioengineering. vol. 11; 2024: 464. 10.3390/bioengineering11050464 PMC11117540 38790331

[68] Sadiq MT, Siuly S, Ur Rehman A, Wang H. Auto-correlation Based Feature Extraction Approach for EEG Alcoholism Identification. In: Health Information Science: 2021// 2021; Cham: Springer International Publishing; 2021: 47–58.

[69] Akbari H, Sadiq MT, Ur Rehman A, Ghazvini M, Naqvi RA, Payan M, Bagheri H, Bagheri H. Depression recognition based on the reconstruction of phase space of EEG signals and geometrical features. Appl Acoust. 2021;179:108078.

[70] Akbari H, Sadiq MT, Jafari N, Too J, Mikaeilvand N, Cicone A, Serra-Capizzano S. Recognizing seizure using Poincaré plot of EEG signals and graphical features in DWT domain. Bratisl Lek Listy. 2023;124(1):12–24. 36519602 10.4149/BLL_2023_002

[71] Sadiq MT, Aziz MZ, Almogren A, Yousaf A, Siuly S, Rehman AU. Exploiting pretrained CNN models for the development of an EEG-based robust BCI framework. Comput Biol Med. 2022;143:105242. 35093844 10.1016/j.compbiomed.2022.105242

[72] Sadiq MT, Yu X, Yuan Z. Exploiting dimensionality reduction and neural network techniques for the development of expert brain–computer interfaces. Expert Syst Appl. 2021;164:114031.

[73] Siuly S, Khare SK, Kabir E, Sadiq MT, Wang H. An efficient Parkinson’s disease detection framework: Leveraging time-frequency representation and AlexNet convolutional neural network. Comput Biol Med. 2024;174:108462. 38599069 10.1016/j.compbiomed.2024.108462

