# Supplementary material for: Microstate permutation complexity of EEG signals distinguishes minimally conscious state plus from minimally conscious state minus
Source: J Neuroeng Rehabil. 2026 Apr 30;23:196. doi: 10.1186/s12984-026-01993-w (PMC13281468; doi:10.1186/s12984-026-01993-w)
Supplement: Supplementary file 1 — Supplementary Material 1. [file 12984_2026_1993_MOESM1_ESM.docx]

**Operational Diagnostic Criteria for VS/UWS, MCS, and MCS+/MCS−, and Key Points for CRS-R Administration**

1. **VS/UWS (vegetative state / unresponsive wakefulness syndrome): operational diagnostic criteria**
   Wakefulness is present (e.g., eye opening and/or sleep–wake cycles), but there is **no reproducible and verifiable** evidence of awareness on standardized bedside examination; specifically, there is no reliable command following, no intelligible verbalization or intentional communication, no functional object use, and no other behaviors clearly indicative of consciousness, with observed behaviors limited to reflexive responses [1].
2. **MCS (minimally conscious state): operational diagnostic criteria**
   MCS is diagnosed when there are **minimal but definite**, albeit inconsistent, signs of awareness, operationally defined by the reproducible observation of at least one of the following: command following; intelligible verbalization; accurate but possibly unstable yes/no responses; or intentional behaviors such as sustained visual pursuit or fixation and context-appropriate emotional responses [2].
3. **MCS− vs. MCS+: operational subcategorization (phenotypic stratification)**
   MCS− is characterized predominantly by nonverbal, lower-level conscious behaviors (e.g., visual pursuit/fixation, localization to noxious stimulation), whereas MCS+ is characterized by higher-level behaviors typically associated with language or symbolic processing, including command following, intelligible speech, or clear intentional communication [3, 4].
4. **Emergence from MCS (eMCS): operational threshold**
   eMCS is operationally defined by the appearance of either **functional communication** (reliable yes/no responses) or **functional object use**, confirmed on repeated assessments [5].
5. **CRS-R (JFK Coma Recovery Scale–Revised): key administration and scoring points**
   CRS-R should be administered after identifying and, where feasible, controlling confounders (e.g., sedative/analgesic effects, metabolic derangements, epileptic activity, pain, and sensory/motor limitations) and in a standardized, low-stimulation environment; arousal facilitation may be used to achieve an optimal level of wakefulness [6]. Critical behaviors should be elicited repeatedly to confirm consistency and goal-directedness; scoring follows the “highest item achieved” principle within each subscale, and diagnostic classification should emphasize the presence of MCS/eMCS-indicative items rather than relying solely on total scores [6]. Because conscious behaviors fluctuate and misdiagnosis risk is substantial, guidelines recommend **serial, multi-time-point CRS-R assessments** with detailed behavioral documentation, and—when uncertainty persists—integration with ancillary tests such as EEG or neuroimaging, while maintaining standardized behavioral assessment as the diagnostic cornerstone [7, 8].

**eMethod**

**Extraction of microstates features**

After obtaining the microstates of the subjects, we extracted three conventional parameters of the corresponding EEG microstates:

（1）Duration: The average time each microstate remains stable.

（2）Coverage: The percentage of time each microstate category covers within all analyzed periods of the EEG segment.

（3）Occurrence: The average number of times each microstate occurs per second.

In our study, we analyzed these three microstate parameters over the entire EEG segment (10 minutes) for each subject.

（4）Transition Probability: The definition of microstate permutation probability involves the frequency of permutations between microstates—that is, the probability that the brain permutes from one microstate to another. Specifically, microstate permutation probability is calculated as the proportion, within a time window, of permutations from a particular microstate (e.g., microstate A) to another specific microstate (e.g., microstate B). It can be used to analyze the dynamic changes of brain functional states, especially in EEG studies of patients with disorders of consciousness, to reveal differences between different states of consciousness.

For example, by setting four different microstates (A, B, C, D), we calculate the probabilities of A permuting to B, A permuting to C, etc., to construct a microstate permutation matrix. This matrix can help researchers quantify the permutation relationships between microstates, thereby further analyzing the spatiotemporal dynamic characteristics of EEG activity.

**Relative Power**

For each patient and channel, we extracted the total power and relative power in five frequency bands of interest: δ (delta) waves (1–4 Hz), θ (theta) waves (4–8 Hz), α (alpha) waves (8–12 Hz), β (beta) waves (12–30 Hz), and γ (gamma) waves (30–45 Hz). For each channel and epoch, the power spectral density was estimated by applying a Fast Fourier Transform (FFT) with a Hamming window. The relative power in each frequency band was defined as the ratio of the total power within that band to the total power in the 1– 45 Hz range.

$$E_{total}=E_{\delta}+E_{\theta}+E_{\alpha}+E_{\beta}+E_{\gamma}$$

Where $E_{i}$ is the energy of band $i$ ($i=\delta,\theta,\alpha,\beta and \gamma$) and $E_{total}$ is the total energy from 0.5 to 45 Hz; and

$${RWE}_{i}=E_{i}/E_{total}$$

Where ${RWE}_{i}$ is the relative wavelet energy of band $i$ ($i=\delta,\theta,\alpha,\beta and \gamma$).

The study by Lechinger et al. [9] showed a significant correlation between frequency band ratios, particularly the ratio of frequencies above 8 Hz to those below 8 Hz, and clinical scores. These ratios provide an intuitive way to assess the balance between higher frequencies (which are likely associated with cognitive activity) and lower frequencies (which are often related to pathological states or drowsiness). Therefore, we also calculated the ratio of Alpha+Beta to Delta+Theta (Alpha+Beta/Delta+Theta) and the ratio of Alpha to Beta (Alpha/Beta).

**Temporal Complexity**

In this study, we used Sample Entropy (SampEn) [10] and Lempel-Ziv Complexity (LZC) [11] to analyze the temporal complexity of EEGs in patients with different levels of consciousness. All features were calculated within the frequency range of 1– 45 Hz.

**LZC**

As a measure of randomness, LZC [12] is related to the number of distinct substrings and their rate of occurrence in the sequence [13]; a higher value corresponds to a higher probability of a new pattern and greater complexity of the dynamic behavior [14]. The measure can directly reflect the physiological information of brain, or information about brain changes caused by brain lesions and the effects of various drugs.

Before calculating LZC, the signal should be converted into a sequence of symbols (0, 1). First, the median value of the signal is used as the threshold, and the gait time series $Y=\{y(1), y(2),\ldots.,y(n)\}$is converted to $P=\{s(1), s(2),\ldots,s(n)\}$, where $s(j)$is expressed as:

|  | $s\left( j \right)=0, x\left( j \right)<T_{d} or s\left( j \right)=1, x\left( j \right)\geq T_{d}$ |  |
| --- | --- | --- |

Let sequence $S$ and sequence $Q$ be two subsequences of $P$, and $\mathrm{SQ}$ is the concatenation sequence of subsequences $S$ and $Q$. When the last character in the $\mathrm{SQ}$ sequence is deleted, the remaining sequence is recorded as $SQ\pi$. Let $v(SQ\pi)$ be the set of all different subsequences in $SQ\pi$. The specific steps are as follows:

1. Set complexity $c(n)=1$, $P=s(1)$,$Q=s(2)$, then $SQ\pi=s(1)$.

2. In general, if $P=s(1), s(2),\ldots,s(r)$，$Q=s(r+1)$, then $SQ\pi=s(1), s(2),\ldots,s(r)$. If $Q\epsilon v(SQ\pi)$, then $Q$ is said to be a subsequence of $SQ\pi$.

3. Update $Q$ to $Q=s(r+1),s(r+2)$, and see if $Q$is in $v(SQ\pi)$.

4. Repeat step (3) until you get a $Q$ sequence that is not in $v(SQ\pi)$. Suppose this process requires $i$ steps. If $Q=s(r+1),s(r+2),s(r+i)$ is not a subsequence of $SQ\pi$ ($SQ\pi=s(1), s(2),\ldots,s(r)$), then increment $c(n)$ by 1.

5. Then, update S to $S=s(1),s(2),\ldots,s(r+i)$, and Q to $Q=s(r+i+1)$.

Repeat the above steps until $Q$ is the last character. In order to obtain a complexity metric $c(N)$ independent of the sequence length, $c(N)$should be normalized by $b\left( n \right)=n/{\log_{2}(n)}$, and $c(N)$ after normalization is:

|  | $LZC= \frac{c(N)}{b(N)}$ |  |
| --- | --- | --- |

**SampEn**

SampEn [15] is used to measure the order of a time series [10]. Similar to ApEn, to compute SampEn, two input parameters should be specified, including a run length m and a tolerance window r. SampEn is the negative natural logarithm of the conditional probability that two sequences similar for m points remain similar at the next point, within a tolerance r, excluding self-matches[16]. Therefore, the measure assigns a non-negative number to a time series, and larger values corresponding to greater signal irregularity.

For a time series of N points, $X\left( n \right)=\{x\left( 1 \right), x\left( 2 \right), \ldots, x\left( N \right)\}$, k variables of length l are formed as $X_{l}\left( k \right)=\{x\left( k+i \right), i=0,\ldots,l-1\}$, and $k=1, \ldots, N-l+1$.The distance between two vectors is calculated as the maximum absolute distance between their corresponding scalar elements. The number of different vectors is counted and normalized as[16]:

|  | $B^{l}\left( r \right)= \frac{1}{N-L}\sum_{i=1}^{N-l} \frac{B_{i}}{N-l-1}$ | (6) |
| --- | --- | --- |

Where $B_{i}$is the number of vectors satisfying the distance less than r.

The above process is repeated until the length l increases to $l+1$, and $B^{l+1}(r)$is obtained. The SampEn can be defined as:

|  | $\mathrm{SampEn}\left( l, r \right)=-ln[\frac{B^{l+1}(r)}{B^{l}(r)}]$ | (7) |
| --- | --- | --- |

**eResults**

**Microstate Parameters**

We compared and examined the microstate parameters between groups.

eFigure 1. Comparisons of Coverage (A), Duration (B), and Occurrence (C) among Four Groups (VS, MCS–, MCS+, CTRL) across the Four Microstates





eFigure 1A, in MS-1 and MS-2, the CTRL group generally showed lower coverage than the other three groups, while it had the highest coverage in MS-3 and MS-4. Specifically, in MS-1, the median of the CTRL group was significantly lower than that of all other groups, with no significant differences among MCS-, MCS+, and VS. In MS-2, the CTRL group remained the lowest, significantly lower than the other three groups, with MCS+ showing relatively higher coverage and MCS- and VS performing similarly. In MS-3, the CTRL group had the highest coverage, significantly higher than the other three groups, with a minimal gap between VS and MCS+ coverage and MCS- slightly exceeding MCS+. By MS-4, overall differences were less pronounced, but the CTRL group’s median remained significantly higher than the other three groups. These results indicate that in most comparisons, particularly during MS-1 and MS-2, the CTRL group differed significantly from the other groups.

eFigure 1B, in MS-1 and MS-2, the CTRL group exhibited shorter durations than the other three groups, whereas it had the longest duration in MS-3, and the VS group showed the longest duration in MS-4. Specifically, in MS-1, the median duration in the CTRL group was significantly lower than that of the other groups, with no significant differences among MCS-, MCS+, and VS. In MS-2, the CTRL group remained the lowest, significantly lower than the other three, while the VS group showed relatively higher duration, and MCS+ durations exceeded those of MCS-. In MS-3, the CTRL group reached the highest coverage, significantly higher than MCS+, with minimal differences among VS, MCS-, and MCS+. Finally, in MS-4, overall differences were less pronounced, but the median duration in the VS group was significantly higher than that in MCS+.

eFigure 1C, in MS-1 and MS-2, the CTRL group exhibited relatively fewer occurrences compared to the other groups, whereas in MS-3 and MS-4, the CTRL group had more occurrences. Specifically, for MS-1, the CTRL group’s occurrences were significantly lower than those of all other groups, with no significant differences observed among VS, MCS-, and MCS+, and a trend of increasing median occurrences per second from VS to MCS+. In MS-2, both MCS- and MCS+ recorded higher occurrences than the CTRL group, reaching significantly higher levels. In MS-3, the CTRL group showed markedly higher occurrences than all other groups, with MCS+ maintaining relatively higher occurrences but differing minimally from VS and MCS-. Finally, in MS-4, the CTRL group continued to exhibit higher occurrences than the other three groups, which did not differ significantly from each other.

**Transition Probability**

**

**

eFigure 2. Comparisons of Transition Probabilities among the Four Groups (VS, MCS–, MCS+, CTRL) for Each Microstate Transition

In eFigure 2, Certain transitions (1→2, 2→1, 3→1, 4→1, 4→2) were lower in the CTRL group than in the other groups, while others (1→3, 2→3, 3→4, 4→3) were higher in CTRL. Specifically, the transition probabilities of 1→4 and 2→3 in CTRL were significantly higher than in MCS+, whereas 3→2 was significantly higher in MCS+ than in CTRL, and 3→4 was significantly higher in VS than in MCS+. Notably, the 4→3 transition stood out in the CTRL group, being significantly higher than in the other groups, highlighting a distinct difference in how frequently this particular state transition occurred.

**Table S1** VS and MCS- Pairwise Mann–Whitney U statistics

| Feature | U | p(uncorrected) | p (Bonferroni-adjusted) | Cliff’s Delta |
| --- | --- | --- | --- | --- |
| Delta | 62 | **0.038088284** | 0.228529704 | -0.448888889 |
| Theta | 153 | 0.097091068 | 0.582546408 | 0.36 |
| Alpha | 166 | **0.027925057** | 0.167550342 | 0.475555556 |
| Beta | 152 | 0.105739202 | 0.634435212 | 0.351111111 |
| Gamma | 131 | 0.455301896 | 1.00 | 0.164444444 |
| (α+β)/(δ+θ) | 166 | **0.027925057** | 0.167550342 | 0.475555556 |
| α/β | 142 | 0.229028967 | 1.00 | 0.262222222 |
| LZC | 60 | **0.031016611** | 0.186099666 | -0.466666667 |
| SampEn | 149 | 0.13538211 | 0.812292660 | 0.324444444 |
| MS-LZC | 143 | 0.213373707 | 1.00 | 0.271111111 |
| MS-PLZC | 70 | 0.081494648 | 0.488968389 | -0.377777778 |

**Table S2** VS and MCS- Classification results of each feature in LOOCV

| Feature | Acc | Sen | Spe | AUC |
| --- | --- | --- | --- | --- |
| Delta | 0.533 | 0.400 | 0.667 | 0.573 |
| Theta | 0.267 | 0.200 | 0.333 | 0.**809** |
| Alpha | 0.633 | 0.**667** | 0.600 | 0.658 |
| Beta | 0.533 | 0.600 | 0.467 | 0.684 |
| Gamma | 0.433 | 0.333 | 0.533 | 0.596 |
| (α+β)/(δ+θ) | 0.533 | 0.600 | 0.467 | 0.551 |
| α/β | 0.500 | 0.400 | 0.600 | 0.591 |
| Power-7 | **0.667** | 0.600 | 0.733 | 0.627 |
| LZC | 0.567 | 0.533 | 0.600 | 0.613 |
| SampEn | 0.633 | 0.**667** | 0.600 | 0.564 |
| NL-2 | 0.467 | 0.400 | 0.533 | 0.538 |
| MS-LZC | 0.533 | 0.533 | 0.533 | 0.564 |
| MS-PLZC | 0.600 | 0.400 | **0.800** | 0.524 |

**Note.** “Positive class: MCS−; Specificity = proportion of correctly identified VS/UWS.”

**Figure S1** VS/UWS vs MCS− LOOCV classification


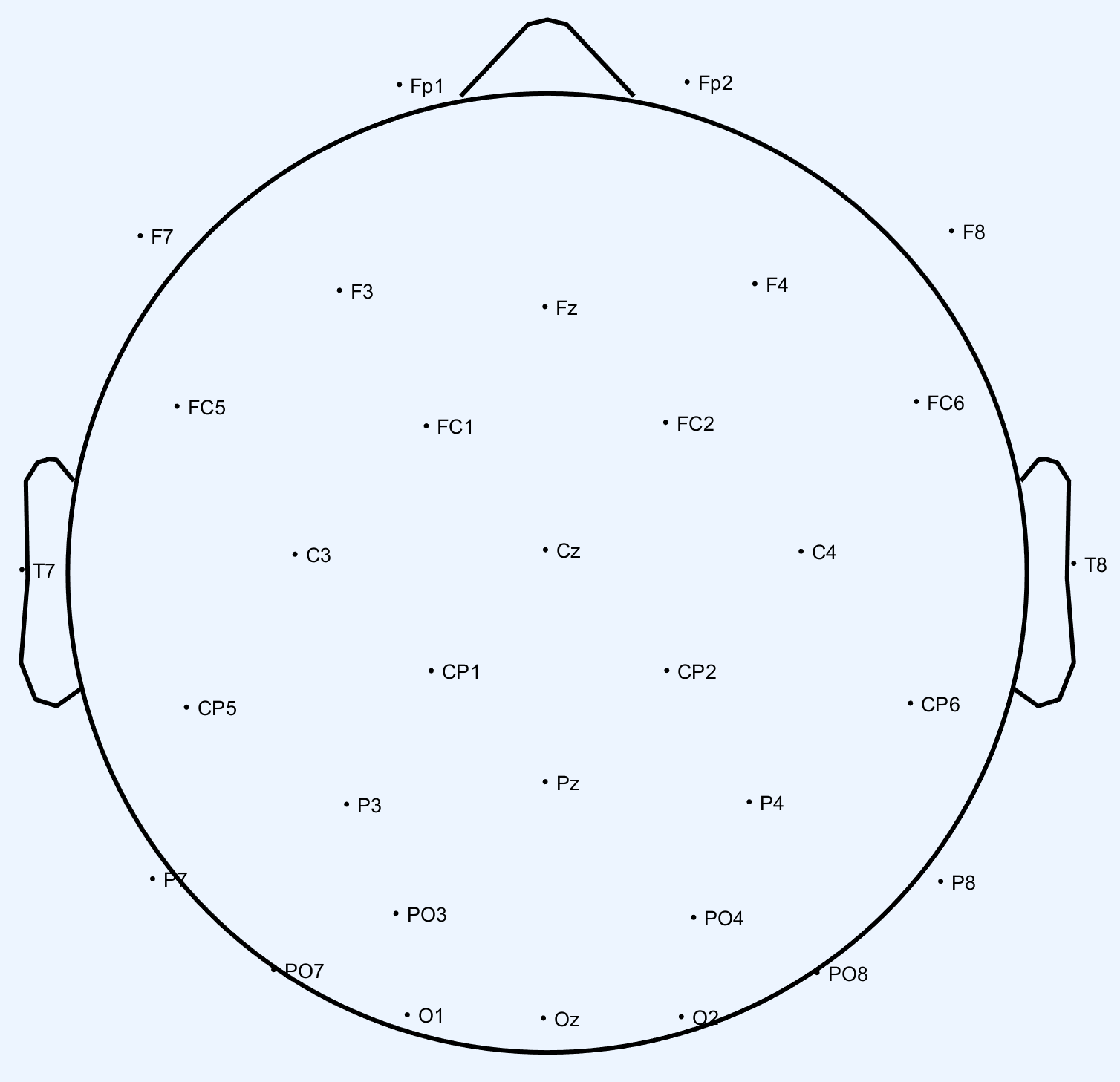


**Figure S2** The electrode layout

**Table S3.** Case-by-case Demographic and Clinical Information

| ID | Age | Sex | Etiology | Time since injury | CRS-R total score |
| --- | --- | --- | --- | --- | --- |
| VS/UWS_01 | 16 | Male | Traumatic brain injury | 2 | 7 |
| VS/UWS_02 | 42 | Male | Stroke | 2 | 2 |
| VS/UWS_03 | 23 | Male | Ischemic–hypoxic | 4 | 5 |
| VS/UWS_04 | 35 | Male | Ischemic–hypoxic | 4 | 5 |
| VS/UWS_05 | 38 | Male | Stroke | 5 | 5 |
| VS/UWS_06 | 52 | Male | Ischemic–hypoxic | 9 | 6 |
| VS/UWS_07 | 23 | Male | Ischemic–hypoxic | 9 | 7 |
| VS/UWS_08 | 55 | Male | Stroke | 3 | 5 |
| VS/UWS_09 | 63 | Male | Stroke | 1.5 | 6 |
| VS/UWS_10 | 34 | Male | Stroke | 36 | 2 |
| VS/UWS_11 | 62 | Male | Ischemic–hypoxic | 1 | 6 |
| VS/UWS_12 | 56 | Male | Stroke | 2 | 7 |
| VS/UWS_13 | 49 | Male | Stroke | 2 | 5 |
| VS/UWS_14 | 37 | Female | Ischemic–hypoxic | 2 | 4 |
| VS/UWS_15 | 62 | Female | Stroke | 2.5 | 4 |
| MCS-_01 | 47 | Male | Stroke | 2 | 8 |
| MCS-_02 | 32 | Male | Stroke | 1 | 7 |
| MCS-_03 | 77 | Female | Ischemic–hypoxic | 1.5 | 10 |
| MCS-_04 | 68 | Female | Ischemic–hypoxic | 1.5 | 9 |
| MCS-_05 | 44 | Male | Ischemic–hypoxic | 1 | 8 |
| MCS-_06 | 44 | Male | Stroke | 2 | 11 |
| MCS-_07 | 42 | Male | Stroke | 2 | 8 |
| MCS-_08 | 66 | Female | Traumatic brain injury | 1 | 6 |
| MCS-_09 | 29 | Female | Ischemic–hypoxic | 3 | 9 |
| MCS-_10 | 24 | Female | Ischemic–hypoxic | 14 | 10 |
| MCS-_11 | 36 | Male | Traumatic brain injury | 5 | 11 |
| MCS-_12 | 49 | Female | Stroke | 9 | 8 |
| MCS-_13 | 15 | Male | Traumatic brain injury | 2.5 | 8 |
| MCS-_14 | 61 | Male | Traumatic brain injury | 5 | 11 |
| MCS-_15 | 56 | Male | Stroke | 3.5 | 8 |
| MCS+_01 | 45 | Female | Traumatic brain injury | 2.5 | 9 |
| MCS+_02 | 44 | Male | Stroke | 1.5 | 10 |
| MCS+_03 | 48 | Male | Traumatic brain injury | 3 | 9 |
| MCS+_04 | 25 | Male | Traumatic brain injury | 5 | 11 |
| MCS+_05 | 51 | Female | Traumatic brain injury | 1 | 12 |
| MCS+_06 | 49 | Female | Traumatic brain injury | 1.5 | 12 |
| MCS+_07 | 61 | Male | Stroke | 2 | 11 |
| MCS+_08 | 68 | Male | Traumatic brain injury | 1.5 | 15 |
| MCS+_09 | 53 | Male | Traumatic brain injury | 6.5 | 10 |
| MCS+_10 | 30 | Female | Ischemic–hypoxic | 132 | 9 |
| MCS+_11 | 18 | Female | Traumatic brain injury | 1 | 9 |
| MCS+_12 | 39 | Male | Stroke | 1.5 | 17 |
| MCS+_13 | 25 | Male | Traumatic brain injury | 1.5 | 13 |
| MCS+_14 | 36 | Male | Stroke | 23 | 9 |
| MCS+_15 | 21 | Male | Traumatic brain injury | 4.5 | 15 |
| CTRL_01 | 48 | Male | None | None | None |
| CTRL_02 | 52 | Female | None | None | None |
| CTRL_03 | 54 | Male | None | None | None |
| CTRL_04 | 46 | Male | None | None | None |
| CTRL_05 | 51 | Male | None | None | None |
| CTRL_06 | 54 | Female | None | None | None |
| CTRL_07 | 50 | Female | None | None | None |
| CTRL_08 | 46 | Male | None | None | None |
| CTRL_09 | 44 | Female | None | None | None |
| CTRL_10 | 37 | Female | None | None | None |
| CTRL_11 | 43 | Female | None | None | None |
| CTRL_12 | 42 | Male | None | None | None |
| CTRL_13 | 46 | Female | None | None | None |
| CTRL_14 | 49 | Male | None | None | None |
| CTRL_15 | 57 | Female | None | None | None |

**Notes:** The unit of “Time since injury” is months.

**eDiscussion**

**Comparison of EEG Relative Power Across Different States of Consciousness**

EEG relative power analysis revealed that the relative power of Delta waves in the VS and MCS groups was significantly higher than that in the CTRL group, which may be associated with thalamic atrophy and a functionally vegetative state. In cases of severe brain injury, Delta waves are often linked to thalamic damage and reduced connectivity between the thalamus and the cortex, potentially leading to a general slowing of cortical activity, a typical feature of diminished consciousness states. Additionally, the power of Beta and Gamma waves was significantly decreased in the VS, MCS-, and MCS+ groups, reflecting impairments in high-frequency activities related to arousal and cognition. This further indicates severe damage to higher cognitive functions and consciousness.

Moreover, Alpha waves are typically associated with relaxed and resting states of the brain. The reduction in Alpha wave power in patients with disorders of consciousness may indicate a decrease in sensory input and internal processing, with significantly weakened spontaneous cortical activity. This finding is consistent with other studies that have observed a positive correlation between levels of consciousness and Alpha activity. In the Beta and Gamma frequency bands, we also observed a significant power reduction in all consciousness disorder groups (VS, MCS-, MCS+) compared to the control group. These frequency bands are generally related to active cognitive processing and information exchange, and their reduced power reflects a decline in cognitive function and consciousness levels. These changes may be associated with a comprehensive weakening of neural network activity, especially in higher-order cognitive and perceptual processes.

Frequency ratio analysis showed that the α+β/δ+θ ratio was lowest in the VS group and highest in the CTRL group, indicating that the balance of cortical activity in patients with impaired consciousness is severely disrupted. A lower ratio suggests dominance of low-frequency activities, further revealing the EEG characteristics of reduced consciousness. These results support the theory of neural network dysfunction in patients with severe brain injuries and further emphasize the importance of EEG spectral analysis in the study of consciousness disorders.

Nonlinear changes in the power spectrum, particularly in the MCS+ group, may reflect transitions in consciousness states, which are related to partial recovery or fluctuations in consciousness. These findings highlight the complexity of brain function following severe injury, suggesting that the process of consciousness recovery may not be linear [17] but involves gradual transitions through complex neural mechanisms, potentially involving interactions among multiple brain regions and networks [18].

**Discussion of Temporal Complexity**

Nonlinear complexity analysis provides important tools for further understanding EEG signal characteristics. In this study, we used SampEn and LZC to evaluate the temporal complexity of EEG signals in patients under different states of consciousness. These measures offer a perspective different from traditional EEG frequency analysis, helping us understand the intrinsic activity patterns of the brain at varying levels of consciousness.

Sample Entropy is primarily used to assess the predictability or regularity of time-series data; lower values typically indicate that the data have higher regularity or predictability. Our study found that the SampEn values in the VS and MCS groups were significantly lower than those in the CTRL. This suggests that in patients with impaired consciousness, the regularity of EEG activity increases, which may be related to simplified or functionally limited neural network activity. Such simplified network activity might be an adaptive adjustment by the brain to maintain basic physiological functions.

**Temporal Complexity**

Temporal complexity reflects the nonlinearity and dynamic characteristics of EEG signals in the temporal dimension, embodying the unpredictability and information richness of the signals. As patients' levels of consciousness improve, brain activity transitions from relatively simple and rigid patterns to more flexible and complex states. For example, in the process from Vegetative State (VS) to Minimally Conscious State (MCS), and then to full recovery, the dynamic regulatory ability of brain neural networks gradually strengthens. The improvement of consciousness levels is usually accompanied by richer information processing and more efficient neural integration. This leads to more complex variation patterns in EEG signals over time, thereby increasing temporal complexity.

The analysis results of Lempel-Ziv Complexity showed that the values in the control group were significantly higher than those in all consciousness-impaired groups. LZC is an important indicator for evaluating the randomness of data; higher LZC values indicate greater randomness and complexity, which is more common in healthy brains. This reflects the efficiency and flexibility of a healthy brain when processing diverse information. The reduction in Lempel-Ziv Complexity further confirms the decreased randomness in the EEG signals of these patients, indicating a decline in the dynamic complexity of brain activity and weakened adaptability.

The results of these nonlinear measures not only show that the complexity of brain activity may gradually increase with partial recovery of consciousness but also display a nonlinear trend of change with alterations in the state of consciousness, with a significant turning point observed in the MCS+ group. This may indicate that as the level of consciousness partially recovers, the activity patterns and complexity of the brain undergo changes. Some studies have shown that consciousness recovery is a nonlinear process, meaning that neural function does not increase proportionally or linearly over time. Instead, neural function may experience slow accumulation or adjustment until reaching a specific critical point (turning point), after which significant progress in neural activity and consciousness recovery occurs.

L. Lei et al. have observed that differences in neural activity between injured and uninjured regions in the spatial dimension follow a non-monotonic pattern of increase. This means that the recovery of neural function between different brain regions is not always synchronous; some damaged regions may require more time to cross the turning point during consciousness recovery, while undamaged regions may show relatively rapid recovery [17] . The turning point observed in the MCS+ group is not only a marker of consciousness recovery in the temporal dimension but also reflects dynamic changes in functional recovery between brain regions. When crossing the turning point, the functional differences between injured and uninjured brain regions may gradually diminish, or the phenomenon of functional imbalance may weaken.

**Traditional EEG Microstate Features Reveal That Healthy Brains Exhibit Greater Efficiency and Flexibility, While DOC Patients Show More Rigid Network Activity**

Overall, CTRL participants displayed lower coverage, shorter duration, and fewer occurrences in MS-1 and MS-2, but higher coverage, longer duration, and more occurrences in MS-3 and MS-4, reflecting greater neural efficiency, flexibility, and capacity for complex processing[19]. In contrast, those with impaired consciousness showed elevated coverage and occurrence in MS-1 and MS-2 or longer durations in MS-4 (notably VS), indicating more rigid or uniform EEG patterns. Additionally, MCS+ exhibited higher coverage in MS-2, suggesting some preserved activity in certain tasks. Transition probabilities further underscored these differences: CTRL rarely transitioned between MS-1 and MS-2, but often shifted into or out of MS-3 and MS-4, whereas patients exhibited deficits in specific transitions, highlighting impaired network functionality[20] and the potential value of microstate analysis in evaluating disorders of consciousness.

1. Laureys S, Celesia GG, Cohadon F, Lavrijsen J, León-Carrión J, Sannita WG, Sazbon L, Schmutzhard E, von Wild KR, Zeman A *et al*: **Unresponsive wakefulness syndrome: a new name for the vegetative state or apallic syndrome**. *BMC Med* 2010, **8**:68.

2. Giacino JT, Ashwal S, Childs N, Cranford R, Jennett B, Katz DI, Kelly JP, Rosenberg JH, Whyte J, Zafonte RD *et al*: **The minimally conscious state: definition and diagnostic criteria**. *Neurology* 2002, **58**(3):349-353.

3. Aubinet C, Larroque SK, Heine L, Martial C, Majerus S, Laureys S, Di Perri C: **Clinical subcategorization of minimally conscious state according to resting functional connectivity**. *Hum Brain Mapp* 2018, **39**(11):4519-4532.

4. Bruno MA, Vanhaudenhuyse A, Thibaut A, Moonen G, Laureys S: **From unresponsive wakefulness to minimally conscious PLUS and functional locked-in syndromes: recent advances in our understanding of disorders of consciousness**. *J Neurol* 2011, **258**(7):1373-1384.

5. Nakase-Richardson R, Yablon SA, Sherer M, Nick TG, Evans CC: **Emergence from minimally conscious state: insights from evaluation of posttraumatic confusion**. *Neurology* 2009, **73**(14):1120-1126.

6. Giacino JT, Kalmar K, Whyte J: **The JFK Coma Recovery Scale-Revised: measurement characteristics and diagnostic utility**. *Arch Phys Med Rehabil* 2004, **85**(12):2020-2029.

7. Giacino JT, Katz DI, Schiff ND, Whyte J, Ashman EJ, Ashwal S, Barbano R, Hammond FM, Laureys S, Ling GSF *et al*: **Practice guideline update recommendations summary: Disorders of consciousness: Report of the Guideline Development, Dissemination, and Implementation Subcommittee of the American Academy of Neurology; the American Congress of Rehabilitation Medicine; and the National Institute on Disability, Independent Living, and Rehabilitation Research**. *Neurology* 2018, **91**(10):450-460.

8. Kondziella D, Bender A, Diserens K, van Erp W, Estraneo A, Formisano R, Laureys S, Naccache L, Ozturk S, Rohaut B *et al*: **European Academy of Neurology guideline on the diagnosis of coma and other disorders of consciousness**. *Eur J Neurol* 2020, **27**(5):741-756.

9. Lechinger J, Bothe K, Pichler G, Michitsch G, Donis J, Klimesch W, Schabus M: **CRS-R score in disorders of consciousness is strongly related to spectral EEG at rest**. *J Neurol* 2013, **260**(9):2348-2356.

10. Richman JS, Moorman JR: **Physiological time-series analysis using approximate entropy and sample entropy**. *Am J Physiol Heart Circ Physiol* 2000, **278**(6):H2039-2049.

11. Ibáñez-Molina AJ, Iglesias-Parro S, Soriano MF, Aznarte JI: **Multiscale Lempel-Ziv complexity for EEG measures**. *Clin Neurophysiol* 2015, **126**(3):541-548.

12. Portnova GV, Tetereva A, Balaev V, Atanov M, Skiteva L, Ushakov V, Ivanitsky A, Martynova O: **Correlation of BOLD signal with linear and nonlinear patterns of EEG in resting state EEG-informed fMRI**. *Frontiers in human neuroscience* 2018, **11**:654.

13. Fatmehsari YR, Bahrami F: **Lempel-Ziv Complexity criteria for nonlinear analysis of gait in patients with Parkinson's disease**. *2011 18th Iranian Conference of Biomedical Engineering (ICBME)* 2011:137-141.

14. Lempel A, Ziv J: **On the complexity of finite sequences**. *IEEE Transactions on information theory* 1976, **22**(1):75-81.

15. Machado C: **CEREBRAL RESPONSE TO PATIENT'S OWN NAME IN THE VEGETATIVE AND MINIMALLY CONSCIOUS STATES**. *Neurology* 2007, **69**(7):708-709.

16. Ruiz-Gómez SJ, Gómez C, Poza J, Gutiérrez-Tobal GC, Tola-Arribas MA, Cano M, Hornero R: **Automated Multiclass Classification of Spontaneous EEG Activity in Alzheimer's Disease and Mild Cognitive Impairment**. *Entropy (Basel)* 2018, **20**(1).

17. Lei L, Liu K, Yang Y, Doubliez A, Hu X, Xu Y, Zhou Y: **Spatio-temporal analysis of EEG features during consciousness recovery in patients with disorders of consciousness**. *Clin Neurophysiol* 2022, **133**:135-144.

18. Rizkallah J, Annen J, Modolo J, Gosseries O, Benquet P, Mortaheb S, Amoud H, Cassol H, Mheich A, Thibaut A *et al*: **Decreased integration of EEG source-space networks in disorders of consciousness**. *NeuroImage: Clinical* 2019, **23**:101841.

19. Trevino G, Lee JJ, Shimony JS, Luckett PH, Leuthardt EC: **Complexity organization of resting-state functional-MRI networks**. *Hum Brain Mapp* 2024, **45**(12):e26809.

20. Baker JT, Dillon DG, Patrick LM, Roffman JL, Brady RO, Jr., Pizzagalli DA, Öngür D, Holmes AJ: **Functional connectomics of affective and psychotic pathology**. *Proc Natl Acad Sci U S A* 2019, **116**(18):9050-9059.
